# Ensemble Optimal Control for Managing Drug Resistance in Cancer Therapies

**DOI:** 10.1007/s11538-026-01661-z

**Published:** 2026-05-21

**Authors:** Alessandro Scagliotti, Federico Scagliotti, Laura Deborah Locati, Federico Sottotetti

**Affiliations:** 1 CIT School, Technical University of Munich, Boltzmannstr. 3/II, Garching bei München, 85748 Germany; 2 Munich Center for Machine Learning (MCML), Munich, Germany; 3 Medical Oncology Unit, Istituti Clinici Scientifici Maugeri IRCCS, Pavia, Italy; 4 Department of Internal Medicine and Medical Therapeutics, University of Pavia, Pavia, Italy

**Keywords:** Ensemble optimal control, Cancer modeling, Drug resistance management, Gradient-based optimization, 49M25, 49M05, 92C50, 49J45

## Abstract

In this paper, we explore the application of ensemble optimal control to derive enhanced strategies for pharmacological cancer treatment, and we tackle the problem of the long-term management of the disease, i.e., when the complete eradication of the tumor is not achievable. In particular, we focus on moving beyond the classical clinical approach of giving the patient the maximal tolerated drug dose (MTD), which does not properly exploit the fight among sensitive and resistant cells for the available resources. Here, we employ a Lotka-Volterra model to describe the competing subpopulations, and we enclose this system within the ensemble control framework. In the first part, we establish general results suitable for application to various cancers. Then, we carry out numerical simulations in the setting of prostate cancer treated with androgen deprivation therapy, yielding a computed policy that is reminiscent of the medical ‘active surveillance’ paradigm. Finally, inspired by the numerical evidence, we propose a variant of the celebrated adaptive therapy (AT), which we call ‘Off-On’ AT.

## Introduction

In this paper, we consider a simple differential model for drug resistance in pharmacological cancer treatments, and we address the uncertainty that affects the dynamics and the Cauchy datum using tools of ensemble control of ODEs.

We recall that, given a time horizon $T>0$, an *ensemble of control systems* in $\mathbb {R}^n$ is a family of controlled ODEs of the form

1.1 $$\begin{aligned} {\left\{ \begin{array}{ll} \dot{x}^\theta (t) = G^\theta \big (t, x^\theta (t), u(t) \big ) & \text{ a.e. } \text{ in } [0,T], \\ x^\theta (0) = x_0^\theta , \end{array}\right. } \end{aligned}$$

where $u:[0,T] \rightarrow \mathbb {R}^m$ is the *m*-dimensional control input, $[0,T]\ni t\mapsto x^\theta (t) \in \mathbb {R}^n$ is the corresponding trajectory, and where the dynamics $G^\theta :[0,T]\times \mathbb {R}^n\times \mathbb {R}^m\rightarrow \mathbb {R}^n$ and the initial condition $x_0^\theta$ depend continuously on the (unknown) *k*-dimensional parameter $\theta$, which varies in a compact set $\Theta \subset \mathbb {R}^k$. In this framework, we aim to find a common policy $t \mapsto u(t)$ for *simultaneously* driving every system of the parametrized family (1.1). This is typically the case when the parameters that appear in the dynamics are subject to statistical errors, and we seek to compute a control that works in every situation (see Ruths and Li 2012). Problems involving ensembles are a timely source of interest for researchers working in Mathematical Control. Indeed, on the one hand, among the recent theoretical contributions in the field, we report (Aronna et al. 2025b; Bettiol and Khalil 2019, 2024) for *averaged* optimal control problems, and Abdel Wahab and Bettiol (2025); Scagliotti (2025) for the *minimax* optimization. Moreover, the Hamilton-Jacobi-Bellman equation related to ensemble control has been considered in Aronna et al. (2026). On the other hand, owing to its flexibility, this framework has found several applications, e.g. in quantum control (Augier et al. 2018; Chittaro and Gauthier 2018; Robin et al. 2022), and in the mathematical modeling of Deep Learning (see Ruiz-Balet and Zuazua 2023; Álvarez-López et al. 2024, 2025; Cipriani et al. 2025) and of Reinforcement Learning (see Murray and Palladino 2018; Pesare et al. 2021), to mention a few. To the best of our knowledge, here we employ for the first time the viewpoint of ensemble control for tuning drug dosage in cancer therapy. Nonetheless, the interplay between optimal control and tumor modeling has a long and glorious story (see the textbook Schättler and Ledzewicz 2015 for an introduction and for a historical overview). Among recent contributions, we refer the reader to Cunningham et al. (2018); Ledzewicz and Schättler (2024), and to Edduweh and Roy (2024) for a Liouville equation for prostate cancer. Moreover, we mention (Aguadé-Gorgorió et al. 2024) for a generalized Lotka-Volterra model for the ecological variety of tumors environment, and Morselli et al. (2023, 2026) for models on oncolytic viruses.

In the first part of the paper (Section 2), we develop in a general setting a framework for ensemble optimal control for cancer therapy. The only major requirement is that the differential model must be affine in the control variable, i.e., it should be an *n*-dimensional system of ODEs of the form

1.2 $$\begin{aligned} {\left\{ \begin{array}{ll} \dot{Z}^\theta (t) = F_0^\theta \big (Z^\theta (t)\big ) + F_1^\theta \big (Z^\theta (t)\big )w(t), \\ Z^\theta (0) = Z_0^\theta , \end{array}\right. } \end{aligned}$$

where $t\mapsto w(t) \in \mathbb R$ is the control that acts on the system, $\theta \in \Theta$ collects the uncertain parameters, and $F_0^\theta , F_1 ^\theta :\mathbb {R}^n\rightarrow \mathbb {R}^n$ are, respectively, the uncontrolled and the controlled part of the dynamics. This setting encloses rather sophisticated multi-species models with secondary resistance mechanisms, like, e.g.,

1.3 $$\begin{aligned} {\begin{matrix} & {\left\{ \begin{array}{ll} \dot{X}_i^\theta (t) = r_i \left( 1- \frac{1}{K} \sum _{j=1}^n X_j^\theta (t) \right) \left( 1-2d_i^I \frac{D(t)}{D_{\max }}\right) X_i^\theta (t) - d_i^T X_i^\theta (t) \\ \qquad \qquad \quad \quad + \sum _{j=1}^n\left( A_{i,j} + A^I_{i,j}\frac{D(t)}{D_{\max }} \right) X_j^\theta (t) \qquad \text{ for } i=1,\ldots ,n, \\ X^\theta _i(0) = f_i N_0 \qquad \qquad \qquad \qquad \qquad \qquad \quad \quad \, \text{ for } i=1,\ldots ,n, \end{array}\right. }\\ & \quad N^\theta (t) = \sum _{i=1}^n X_i^\theta (t),\\ & \quad \theta = \left( (r_i)_{i}, K, (d^I_i)_{i}, (d^T_i)_{i}, (A_{i,j})_{i,j} , (A_{i,j}^I)_{i,j}, (f_i)_i, N_0 \right) \in \Theta , \end{matrix}} \end{aligned}$$

where $t\mapsto D(t)$ is the control and represents the dose of the drug given to a patient, and $t\mapsto X_i^\theta (t)$ is the function describing the size of the *i*-th cancer sub-population as the time *t* varies (see also Table 1).

**Table 1:** Model parameters and variables for the system in eq. (1.3).

**Parameter**	**Meaning**	**Units**
$X_i(t)$	Size of subpopulation *i*	cells
*N*(*t*)	Total population $\sum _{i=1}^n X_i(t)$	cells
$r_i$	Proliferation rate of subpopulation *i*	$\hbox {time}^{-1}$
*K*	Carrying capacity	cells
$d_i^T$	Turnover (natural death) rate	$\hbox {time}^{-1}$
$d_i^I$	Drug sensitivity coefficient of subpopulation *i*	dimensionless
$A_{i,j} \ i\ne j$	Spontaneous transition rate $j \rightarrow i$	$\hbox {time}^{-1}$
$A_{i,i} \le 0$	Spontaneous ‘evolution rate’ of subpopulation *i*	$\hbox {time}^{-1}$
$A^I_{i,j} \ i\ne j$	Drug-induced transition rate $j \rightarrow i$	$\hbox {time}^{-1}$
$A_{i,i}^I \le 0$	Drug-induced ‘evolution rate’ of subpopulation *i*	$\hbox {time}^{-1}$
*D*(*t*)	Drug dosage (control)	dosage
$D_{\max }$	Maximum drug dosage	dosage
$f_i$	Initial fraction of subpopulation *i*	dimensionless
$N_0$	Initial total population	cells

In fact, we can consider even more refined models than the one in (1.3), where the drug concentration around the cancer cells does coincide with the drug dose. In a more realistic description, concentration $t\mapsto C(t)$ and dose $t\mapsto D(t)$ can be related via a differential equation, such as, e.g.,

$$\begin{aligned} \dot{C}(t) = -k^C C(t) + k^I \frac{D(t)}{D_{\max }} . \end{aligned}$$

In this case, we set $Z:=(C,X_1,\ldots ,X_n)$ and we modify accordingly (1.3) so that the induced death rate and the inter-species evolution is triggered by *C*(*t*) in place of *D*(*t*). Moreover, a direct computation shows that $t\mapsto Z(t)$ solves as well a control-affine system of the form (1.2), where $t\mapsto D(t)$ is once again the control input. We insist on the fact that the affine dependence in the control variable is crucial for our analysis—in particular, for the results contained in Theorem 2.2. Once a suitable control-affine model has been selected, the ensemble optimal control approach consists in finding a drug administration policy $t\mapsto D(t)$ that takes into account the uncertainty that affects *(some of)* the model parameters. The goal of this paper is to propose strategies that exhibit enhanced performances in the long-term disease management. In particular, we focus on moving beyond the classical clinical approach of giving to the patient the maximal tolerated dose (MTD), which consists in setting ${D}_{\textrm{MTD}}(t) = {D_{\max }}$ for every $t>0$ in eq. (1.3). When sensitive and resistant clones compete for the available resources—as in solid cancers^1^—, MTD has already been shown to be sub-optimal. Indeed, it leads to the extinction of the sensitive cells and to the emerging of a resistant population (Silva et al. 2012). Here, we aim to obtain a policy of drug dosage by solving an ensemble optimal control problem over a fixed time horizon [0, *T*], where *T* is pre-determined (see Remark 8 for the choice in our simulations). To do this, the key-steps are the definition of a compact set $\Theta$ where the uncertain parameters $\theta$ vary, and the introduction of a probability measure $\mu \in \mathcal {P}(\Theta )$ that describes our knowledge about the distribution of $\theta$. For instance, one may consider repeated measurements of tumor burden in a cohort of patients over the course of therapy, where treatment interruptions (vacations) are employed. Model parameters for each patient could then be estimated via standard parameter identification procedures (see, e.g., Nelles 2020). The resulting collection of estimates $\theta _1,\ldots ,\theta _k$ may be interpreted as independent samples from the underlying distribution, yielding the definition of an empirical probability measure $\mu :=\frac{1}{k} \sum _{j=1}^k \delta _{\theta _j}$, which can be used to study new or existing treatment schedules through ensemble optimal control. Hence, in this framework, the dosage strategy is computed through the minimization of a functional $\mathcal {J}:\mathcal {U}\rightarrow \mathbb {R}$ of the form

1.4 $$\begin{aligned} \mathcal {J}({D}) :=\int _\Theta \int _0^T \ell \left( {N_D^\theta (t)} \right) \,\textrm{d}{t} \,\textrm{d}\mu (\theta ), \end{aligned}$$

where $\mathcal {U}:=\{{D} \in L^2([0,T],\mathbb {R}): 0\le {D(t)}\le {D_{\max }} \, \text{ for } \text{ a.e. } {t}\}$, ${N_D^\theta (t)}$ is derived from eq. (1.3), and $\ell :\mathbb {R}\rightarrow \mathbb {R}$ is a proper penalization cost on the tumor size. For the problem of minimizing (1.4), we provide a $\Gamma$-convergence result (see Theorem 2.2), and we provide the explicit expression for the projected gradient field (Proposition 2.5 and 2.8), moving beyond (Scagliotti 2023a, b), where the control variable was unconstrained. Finally, we observe that, roughly speaking, minimizing (1.4) means that we look for a control that ‘does *on average* a good job on the elements of the ensemble’. In this regard, we point out that, even though a *minimax* ensemble control formulation is a viable option (see Scagliotti 2025), in the application that we are considering here it is rather distant from the clinical practice. Indeed, the focus on the improvement of the least favorable cases usually comes at the expenses of a performance degradation on the most likely ones.

In the second part of the paper, we study a simplified system that describes the dynamics of the two subpopulations of a tumor (sensitive and resistant) when the patient is undergoing a pharmacological treatment. Following (Cunningham et al. 2018; Strobl et al. 2021), in the present analysis we assume that the resistant subpopulation consists of descendants of pre-existing clones, and we do not include a mechanism of acquired (secondary) resistance. Namely, adopting the same notations as in Strobl et al. (2021), we render the competition for the limited resources through a Lotka-Volterra system, and we address the following ODE in $\mathbb {R}^2$:

1.5 $$\begin{aligned} {\begin{matrix} & {\left\{ \begin{array}{ll} \dot{S}(t) = r_S\left( 1 - \frac{S(t)+R(t)}{K} \right) \left( 1 - 2d_D\frac{D(t)}{D_{\max }} \right) S(t) - d_T S(t), \\ \dot{R}(t) = r_R\left( 1 - \frac{S(t)+R(t)}{K} \right) R(t) - d_T R(t), \end{array}\right. } \\ & \quad N(t) :=S(t) + R(t), \end{matrix}} \end{aligned}$$

where the time-dependent functions $t\mapsto S(t)$ and $t\mapsto R(t)$ denote, respectively, the amount of cells of the tumor population at the instant *t* that are sensitive and resistant to a certain drug, while $t\mapsto N(t)$ accounts for the total population (see also Table 2). In eq. (1.5) the control is $t\mapsto D(t)$, which takes value in $[0,D_{\max }]$ and represents the drug concentration in the tumor environment. In our model, we assume that the fraction of cells killed by the drug is linearly proportional to the given dose. This classical hypothesis, formulated in Norton and Simon (1977), has been widely adopted in the literature (see, e.g., Schättler and Ledzewicz 2015; Strobl et al. 2021).

**Table 2:** Model parameters and variables for the simplified two-population model in eq. (1.5).

**Parameter**	**Meaning**	**Units**
*S*(*t*)	Size of sensitive subpopulation	cells
*R*(*t*)	Size of resistant subpopulation	cells
*N*(*t*)	Total population $S(t)+R(t)$	cells
$r_S$	Proliferation rate of sensitive cells	$\hbox {time}^{-1}$
$r_R$	Proliferation rate of resistant cells	$\hbox {time}^{-1}$
*K*	Carrying capacity	cells
$d_T$	Turnover (natural death) rate	$\hbox {time}^{-1}$
$d_D$	Drug sensitivity coefficient (sensitive cells)	dimensionless
*D*(*t*)	Drug dosage (control)	dosage
$D_{\max }$	Maximum drug dosage	dosage

In Section 3 we take advantage of the parameters estimates reported in Strobl et al. (2021) and we formulate an ensemble optimal control problem for *prostate cancer* (non-metastatic and castration sensitive, m0CSPC) *treated with androgen deprivation therapy (ADT)*. We compare the performances using the ‘time to progression’ of the disease (TTP), and we adopt the Adaptive Therapy (AT) proposed in Gatenby et al. (2009); Zhang et al. (2017) and MTD as benchmark strategies. When the integral cost $\ell$ is linear, the resulting optimal strategy turns out to behave closely to the MTD protocol. However, when $\ell$ has a hyperbolic profile, the computed policy suggests a delayed starting of the therapy, and on average it outperforms MTD and AT in terms of ‘time to progression’ (see Tables 4 and 6). Moreover, this strategy is reminiscent of medical paradigm known as ‘active surveillance’ (Karim et al. 2025; Preisser et al. 2024). While this feature may be of conceptual interest, a significant limitation of the computed policy is that, during the initial phase, it permits substantial tumor growth, which may limit its practical applicability. Finally, to amend this point, we propose a variant of the AT that we call ‘Off-On’ Adaptive Therapy, which combines the ‘active surveillance’ paradigm (i.e., delayed starting of the therapy) with adaptive periods of treatment vacation (Gatenby et al. 2009; Zhang et al. 2017), showing promising results (see Table 7).

## Ensemble Optimal Control Formulation: Theoretical Framework

In this section, we perform a general analysis for the ensemble control problems formulated in eq. (1.3). First, we show how to enclose eq. (1.3) within the theoretical framework of ensembles of control-affine systems tackled in Scagliotti (2023b, 2025). Then, we define the ensemble optimal control problem, and we show that it admits minimizers and that a $\Gamma$-convergence result holds. Finally, we study the gradient of the functional involved in the optimization problem.

### Definition of the Dynamics

We recall that an ensemble of control-affine systems has the form $\dot{X}^\theta = \tilde{F}_0^\theta (X^\theta ) + \tilde{F}_1^\theta (X^\theta )u$. In order to identify the drift $\tilde{F}_0^\theta$ and the controlled vector field $\tilde{F}_1^\theta$, we set $\theta :=\left( (r_i)_{i}, K, (d^I_i)_{i}, (d^T_i)_{i}, (A_{i,j})_{i,j}, (A_{i,j}^I)_{i,j}, (f_i)_i, N_0 \right) \in \Theta$, and we rearrange eq. (1.3) as follows:

2.1 $$\begin{aligned} {\begin{matrix} & \frac{\textrm{d}}{\textrm{d}t} X_D^\theta (t)= \frac{\textrm{d}}{\textrm{d}t} \left( X_{D\ i}^\theta (t) \right) _{i=1,\ldots ,n} = \tilde{F}_0^\theta \left( X_D^\theta (t)\right) + \tilde{F}_1^\theta \left( X_D^\theta (t)\right) D(t) \\ & = \left( { \textstyle r_i \left( 1- \frac{1}{K} \sum _{j=1}^n X_j^\theta (t) \right) X_i^\theta (t) - d_i^T X_i^\theta (t) + \sum _{j=1}^n A_{i,j} X_j^\theta (t) } \right) _{i=1,\ldots ,n}\\ & \quad + \left( { \textstyle -r_i \left( 1- \frac{1}{K} \sum _{j=1}^n X_j^\theta (t) \right) \frac{2d_i^I}{D_{\max }} X_i^\theta (t) + \sum _{j=1}^n\frac{A^I_{i,j}}{D_{\max }} X_j^\theta (t) } \right) _{i=1,\ldots ,n} D(t), \end{matrix}} \end{aligned}$$

with the initial condition

2.2 $$\begin{aligned} X^\theta _{D\ i}(0) = f_i N_0 \qquad i=1,\ldots ,n, \end{aligned}$$

where $N_0\in [0,K]$ denotes the initial size of the tumor. We briefly discuss below the role of the parameters appearing in eq. (2.1):

- $r_i>0$ denotes the reproduction rate of the *i*-th subpopulation, for $i=1,\ldots ,n$;
- $K, N_0$ denote, respectively, the carrying capacity of the system and the total initial population of cancer cells;
- $d_i^I\ge 0$ denotes the drug sensitivity coefficient of the *i*-th subpopulation, for $i=1,\ldots ,n$. Without loss of generality, we can assume that $d_1^I\ge d_2^I\ge \ldots \ge d_n^I\ge 0$;
- $d_i^T\ge 0$ denotes the turnover rate of the *i*-th subpopulation, for $i=1,\ldots ,n$;
- $f_i\ge 0$ denotes the fraction of the initial total population $N_0$ that belongs to the *i*-th subpopulation, for $i=1,\ldots ,n$;
- $A_{i,j}\ge 0$ denotes the ‘spontaneous’ rate of the evolution (in the Darwinian sense) of the *j*-th subpopulation into the *i*-th subpopulation, for $i,j=1,\ldots ,n$ and $i\ne j$;
- $A_{i,j}^I\ge 0$ denotes the drug-induced rate of the evolution (in the Darwinian sense) of the *j*-th subpopulation into the *i*-th subpopulation, for $i,j=1,\ldots ,n$ and $i\ne j$;
- $A_{i,i}\le 0$ and $A^I_{i,i}\le 0$ denote, respectively, the ‘spontaneous’ and drug-induced rate of the *i*-th subpopulation with which it evolves into some other species (‘evolution rate’).

We observe that the condition $d_i^I=0$ implies that the *i*-th population is completely insensitive to the drug. We insist on the fact that, in order enforce that the inter-species evolution does not increase the total population, we require the following constraints on $(A_{i,j})_{i,j}, (A_{i,j}^I)_{i,j}$:

2.3 $$\begin{aligned} A_{i,i} + \sum _{j\ne i}A_{j,i}=0, \qquad A_{i,i}^I + \sum _{j\ne i}A_{j,i}^I=0, \end{aligned}$$

together with the sign constraints $A_{i,i}\le 0$ and $A^I_{i,i}\le 0$, and $A_{i,j}\ge 0$ and $A_{i,j}^I\ge 0$ for $i\ne j$. Moreover, we assume that $K\in [K_{\min },K_{\max }]$ and $N_0\in [0,K]$, and that $\sum _{i=1}^n f_i=1$. We observe that

2.4 $$\begin{aligned} \big ( X_{D\, i}(0)^\theta \big )_{i=1,\ldots ,n} \in \Delta ^\theta :=\left\{ X \in \mathbb {R}^n : X_i \ge 0 \ \forall i, \, 0\le \sum _{i=1}^n X_i \le K \right\} \end{aligned}$$

for every $\theta \in \Theta$, and we introduce

2.5 $$\begin{aligned} \Delta :=\left\{ X \in \mathbb {R}^n : X_i \ge 0 \ \forall i, \, 0\le \sum _{i=1}^n X_i \le K_{\max } \right\} , \end{aligned}$$

which satisfies $\Delta \supset \Delta ^\theta$ for every $\theta \in \Theta$. Given $T>0$, we consider as the space of admissible controls

2.6 $$\begin{aligned} \mathcal {U}:=\{ {D} \in L^2([0,T],\mathbb {R}): 0\le {D(t)\le D_{\max } } \, \text{ for } \text{ a.e. } t \}. \end{aligned}$$

We notice that $\mathcal {U}$ is a convex subset of $L^2([0,T],\mathbb {R})$, which we equip with the usual Hilbert space structure. We first show that the simplex $\Delta$ defined above is invariant for the dynamics that we are considering.

#### Lemma 2.1

Let us consider ${D}\in \mathcal {U}$ defined as in eq. (2.6), and the simplex $\Delta \in \mathbb {R}^{n}$ introduced in eq. (2.5). For every $\theta \in \Theta$, if ${X_D^\theta (0)}\in \Delta ^{\theta }$, then ${X_D^\theta (t)} \in \Delta ^{\theta }$ (and, in particular, $X_D^\theta (t) \in \Delta$) for every $t \in [0,T]$.

#### Proof

See Appendix A. $$\square $$

Unfortunately, the vector fields $\tilde{F}_0^\theta ,\tilde{F}_1^\theta :\mathbb {R}^{n} \rightarrow \mathbb {R}^{n}$ involved in the differential model (2.1) do not satisfy the usual working assumptions, which require the fields to be globally Lipschitz continuous and to have a sub-linear growth (see, e.g., Scagliotti 2025, Hypothesis 2.1). However, Lemma 2.1 enables us to circumvent this issue. Indeed, we can define the vector fields $F_0^\theta ,F_1^\theta :\mathbb {R}^{n} \rightarrow \mathbb {R}^{n}$ as follows:

2.7 $$\begin{aligned} F_0^\theta ({X}) :=\rho ({X})\tilde{F}_0^\theta ({X}) \quad \text{ and } \quad F_1^\theta ({X}) :=\rho ({X}) \tilde{F}_1^\theta ({X}) \end{aligned}$$

for every ${X} \in \mathbb {R}^{n}$ and for every $\theta \in \Theta$, where $\rho :\mathbb {R}^{n}\rightarrow [0,1]$ is a smooth cut-off function such that $\rho \equiv 1$ in $B_{{2K_{\max }}}(0)$ and $\textrm{supp}{(\rho )}\subset B_{{3K_{\max }}}(0)$. On the one hand, considering $F_0^\theta ,F_1^\theta$ in place of $\tilde{F}_0^\theta ,\tilde{F}_1^\theta$ does not alter the trajectories that are of interest for our model, owing to Lemma 2.1 and since $\Delta \subset B_{{2K_{\max }}}(0)$. On the other hand, $F_1^\theta , F_2^\theta$ fall perfectly within the classical theoretical framework of ensembles of control-affine systems.

### Ensemble Optimal Control Problem and Related Functional

We shall propose a therapy schedule resulting in the (approximate) solution of an ensemble optimal control problem. To this end, we assume that we are given a probability measure $\mu \in \mathcal {P}(\Theta )$ on the space of the unknown parameters $\theta$ of the control system in eq. (2.1) and (2.2). Throughout the paper, the space $\Theta \subset \mathbb {R}^{m}$ is assumed to be compact. Recalling the definition of $\mathcal {U}$ in eq. (2.6), we consider the ensemble optimal control problem associated to the functional $\mathcal {J}:\mathcal {U}\rightarrow \mathbb {R}$ defined as

2.8 $$\begin{aligned} \mathcal {J}({D}) :=\int _\Theta \int _0^T \ell \left( {N_D}^\theta ({t} )\right) \,\textrm{d}{t} \,\textrm{d}\mu (\theta ), \end{aligned}$$

where $N_D^\theta (t)=\sum _{i=1}^nX_{D\ i}^\theta (t)$, with $X_{D\, 1}^\theta , \ldots , X_{D\, n}^\theta$ solutions of eq. (3.3) corresponding to the admissible control ${D}\in \mathcal {U}$ and to the parameters $\theta \in \Theta$, and where $\ell :\mathbb {R}\rightarrow \mathbb {R}$ is a cost function of class $C^2$.

#### Remark 1

The function that we integrate in eq. (2.8) depends *on the total cancer population* ${N_D^\theta (t)}$ *at every instant* $t\in [0,T]$. This choice has a precise interpretation in the model. Indeed, in a real-world scenario, it is not realistic to have access separately to the sizes of the *n* different subpopulations.

#### Remark 2

The functional $\mathcal {J}$ introduced in eq. (2.8) designs an *averaged* ensemble optimal control problem, as we saturate the dependence on the parameter $\theta$ by averaging with respect to the probability measure $\mu$. An alternative paradigm consists in optimizing with respect to the worst-case scenario. Namely, this would result in addressing the minimax problem induced by the functional

2.9 $$\begin{aligned} \mathcal {J}_{\max } :=\max _{\theta \in \Theta } \left( \int _0^T \ell \left( {N_D}^\theta ({t} )\right) \,\textrm{d}{t} \right) , \end{aligned}$$

recently studied in Scagliotti (2025). However, in the medical application that we are considering in the present paper, a minimax formulation can lead to over-conservative strategies. Indeed, it is more natural—and closer to the clinical practice—to design treatment strategies that are effective for the majority of the patients, rather than trying to achieve the best possible outcome on the worst-cases. This is due to the fact that the improvement on the least favorable systems of the ensemble usually comes at the expenses of a performance degradation on the most likely ones (see Scagliotti 2025, Section 6). Nevertheless, for comparison, in Section 3 we include numerical simulations addressing the minimax ensemble control problem.

Below, we show that the ensemble optimal control problem consisting in minimizing $\mathcal {J}$ admits solution. Moreover, using the tools of $\Gamma$-convergence, we establish a ‘stability result’ for the minimizers of $\mathcal {J}$ with respect to perturbations in $\mu$. Given a sequence $(\mu _{k})_{{k}\ge 1}\in \mathcal {P}(\Theta )$, we write $\mu _{k}\rightharpoonup ^* \mu$ as ${k}\rightarrow \infty$ to denote the weak-$*$ convergence of probability measures, i.e.,

$$\begin{aligned} \lim _{{k}\rightarrow \infty } \int _\Theta \phi (\theta )\,\textrm{d}\mu _{k}(\theta ) = \int _\Theta \phi (\theta )\,\textrm{d}\mu (\theta ) \end{aligned}$$

for every $\phi :\Theta \rightarrow \mathbb {R}$ bounded and continuous.

#### Theorem 2.2

The functional $\mathcal {J}:\mathcal {U}\rightarrow \mathbb {R}$ defined in eq. (2.8) admits minimizer in $\mathcal {U}$.

Moreover, given a sequence $(\mu _{k})_{{k}\ge 1}\in \mathcal {P}(\Theta )$ such that $\mu _{k}\rightharpoonup ^* \mu$ as ${k}\rightarrow \infty$, if we define

$$\begin{aligned} \mathcal {J}_{k}({D}) :=\int _\Theta \int _0^T \ell \left( { N_D^\theta (t )}\right) \,\textrm{d}t \,\textrm{d}\mu _{k}(\theta ) \end{aligned}$$

for every ${D}\in \mathcal {U}$ and for every ${k}\ge 1$, and if we denote with ${D}^\star _{k}$ a minimizer of $\mathcal {J}_{k}$ for every ${k}\ge 1$, then we have $\min _\mathcal {U}\mathcal {J}= \lim _{{k}\rightarrow \infty }\mathcal {J}_{k}({D}^\star _{k})$, and every $L^2$-weak limiting point of $({D}^\star _{k})_{{k}\ge 1}$ is a minimizer for $\mathcal {J}$.

#### Proof

The proof of the first part follows using the direct method of the Calculus of Variations, considering the weak topology of $L^2$. More precisely, the lower semi-continuity of $\mathcal {J}$ is contained as a particular case of the proof of (Scagliotti 2023b, Theorem 3.2). For the weak coercivity, we recall that the space of admissible controls $\mathcal {U}$ is strongly closed and convex, hence it is weakly closed. Moreover, it is contained in the ball of $L^2$ centered at the origin and with radius $\sqrt{T}$. Therefore, the whole domain $\mathcal {U}$ of $\mathcal {J}$ is weakly compact, and $\mathcal {J}$ admits minimizer. The second part part of the statement descends from the fact that the sequence of functionals $(\mathcal {J}_{k})_{{k}\ge 1}$ is $\Gamma$-convergent to the functional $\mathcal {J}$ with respect to the $L^2$-weak topology (see Scagliotti 2023b, Theorem 4.6). Hence, observing that the functionals $(\mathcal {J}_{k})_{{k}\ge 1}$ are defined on the same weakly compact domain $\mathcal {U}$, the thesis follows by using (Scagliotti 2023b, Corollary 4.8). $\square$

#### Remark 3

The second part of Theorem 2.2 plays a pivotal role in the applications. Indeed, each evaluation of the functional $\mathcal {J}$ requires the computation of the trajectories $t\mapsto {X_D}^\theta (t)$ solving eq. (2.1) and (2.2) for every $\theta \in \textrm{supp}(\mu )$. When the support of the measure $\mu \in \mathcal {P}(\Theta )$ contains infinitely many elements, this turns out to be completely impractical. In the case we approximate $\mu$ with a discrete measure $\mu _{k}$ with finite support, Theorem 2.2 ensures that any minimizer of $\mathcal {J}_{k}$ is a good competitor as well for the original objective functional $\mathcal {J}$. Finally, we observe that the construction of discrete approximating measures is an active research field (see, e.g., Mérigot et al. 2021; Auricchio et al. 2024; Auricchio and Zhang 2024; Auricchio et al. 2024)

#### Remark 4

We recall that the Pontryagin Maximum Principle (PMP) provides necessary conditions for optimal controls (see, e.g., Bressan and Piccoli 2007, Section 6 for a general introduction). In the framework of averaged ensemble optimal control, the PMP has been studied in Bettiol and Khalil (2019), and it provides insights on the structure of minimizers. More precisely, in our setting where the cost does not explicitly depend on the control input, from (Bettiol and Khalil 2019, Theorem 3.3) it follows that, for every $\mu \in \mathcal {P}(\Theta )$, any optimal control $u^\star \in \arg \min _\mathcal {U}\mathcal {J}$ satisfies the following condition:

2.10 $$\begin{aligned} D^\star (t) \in \arg \max _{v\in [0,D_{\max }]} \left\{ \int _{\Theta } g^\theta _{D^\star }(t) \cdot F_1^\theta \big ( X_{D^\star }^\theta (t) \big ) v \,\textrm{d}\mu (\theta ) \right\} \end{aligned}$$

for a.e. $t\in [0,T]$, where $t\mapsto g^\theta _{D^\star }(t)$ solves the *backward adjoint equation*

$$\begin{aligned} {\left\{ \begin{array}{ll} \dot{g}_{D^\star }^\theta (t) = -\ell ' \left( N^\theta _{D^\star }(t) \right) (1,1) -g_{D^\star }^\theta (t) \left( \frac{\partial F_0^\theta (X_{D^\star }^\theta (t))}{\partial x} + \frac{\partial F_1^\theta (X_{D^\star }^\theta (t))}{\partial x} D^\star (t) \right) ,\\ g_{D^\star }^\theta (T) = (0,\ldots ,0), \end{array}\right. } \end{aligned}$$

for every $\theta \in \Theta$. We notice that, in the present setting, we only have normal extremals, as we do not impose any terminal-state constraint. From eq. (2.10), we deduce the *bang-bang* relation

$$\begin{aligned} D^\star (t) = {\left\{ \begin{array}{ll} D_{\max } & \text{ if } \psi (t)>0, \\ 0 & \text{ if } \psi (t)<0, \end{array}\right. } \end{aligned}$$

where $\psi (t) :=\int _{\Theta } g^\theta _{D^\star }(t) \cdot F_1^\theta \big ( X_{D^\star }^\theta (t) \big ) \,\textrm{d}\mu (\theta )$. If the set $\{ t \in [0,T]: \psi (t) =0 \}$ has positive Lebesgue measure, the control $D^\star$ is said to be a *singular arc*. For more details on singular arcs in averaged ensemble optimal control, we refer to Aronna et al. (2025a).

Finally, it is worth mentioning that, in the case of the minimax optimal control problem related to the minimization of eq. (2.9), the necessary optimaltiy condition for $D^\star _{\textrm{mm}} \in \arg \min _\mathcal {U}(\mathcal {J}_{\max })$ looks rather similar to eq. (2.10). Namely, we have that there exists $\bar{\mu }_{\max } \in \mathcal {P}(\Theta )$ such that

2.11 $$\begin{aligned} D^\star _{\textrm{mm}} (t) \in \arg \max _{v\in [0,D_{\max }]} \left\{ \int _{\Theta } g^\theta _{D^\star _{\textrm{mm}}}(t) \cdot F_1^\theta \big ( X_{D^\star _{\textrm{mm}}}^\theta (t) \big ) v \,\textrm{d}\bar{\mu }_{\max }(\theta ) \right\} \end{aligned}$$

for a.e. $t\in [0,T]$. We insist on the fact that $\bar{\mu }_{\max }$ is not given a priori, and it is in general hard to identify. For more details, see (Vinter 2005) and (Scagliotti 2025, Remark 5.10). In view of the analogy between eq. (2.11) and (2.10), we expect $D^\star _{\textrm{mm}}$ to exhibit as well *bang-bang* behavior.

### Gradient of the Objective Functional

In this part, we carry out the computations for the gradient of the functional $\mathcal {J}$ that we aim at minimizing. We report that the findings that we show below can be applied as well when we deal with an approximated functional $\mathcal {J}_{k}$ related to a measure $\mu _{k} \approx \mu$. A main difficulty arises from the fact that admissible controls are constrained to the interval $[0,D_{\max }]$, so that the control space is convex but not linear; to address this, we develop here a careful argument.

For this reason, it is convenient to introduce the functional $\mathcal {J}':L^2([0,T], \mathbb {R})\rightarrow \mathbb {R}$ defined according to eq. (2.8) *for every* ${D} \in L^2([0,T], \mathbb {R})$. More precisely, we set

2.12 $$\begin{aligned} \mathcal {J}'({D}):=\int _\Theta \int _0^T \ell \left( {N_D^\theta (t)}\right) \,\textrm{d}{t} \,\textrm{d}\mu (\theta ), \end{aligned}$$

where $N_D^\theta (t)=\sum _{i=1}^nX_{D\ i}^\theta (t)$, with $X_D^\theta = \left( X_{D\ 1}^\theta , \ldots , X_{D\ n}^\theta \right)$ solving

2.13 $$\begin{aligned} \frac{\textrm{d}}{\textrm{d}{t}} {X_D^\theta }({t}) = F_0^\theta \left( {X_D^\theta } ({t}) \right) + F_1^\theta \left( {X_D^\theta } ({t}) \right) {D}({t}), \qquad {X_{D\ i}}^\theta (0) = {f_i N_0} \end{aligned}$$

for every $\theta \in \Theta$. In other words, $\mathcal {J}'$ is the extension of $\mathcal {J}$ (which is defined only on $\mathcal {U}$) to the whole $L^2$, as illustrated in the next lemma. We observe that, in order to ensure that $\mathcal {J}'$ is well-defined for every ${D}\in L^2([0,T], \mathbb {R})$, it is crucial to consider the truncated vector fields $F_0^\theta , F_1^\theta$ introduced in eq. (2.7) in place of $\tilde{F}_0^\theta , \tilde{F}_1^\theta$.

#### Lemma 2.3

Let $\mathcal {J}:\mathcal {U}\rightarrow \mathbb {R}$ and $\mathcal {J}':L^2([0,T],\mathbb {R})\rightarrow \mathbb {R}$ be defined as in eq. (2.8) and eq. (2.12), respectively. If ${X_D^\theta }(0) = \left( {f_1N_0,\ldots , f_nN_0} \right) \in \Delta ^{\theta }$ for every $\theta \in \Theta$, then $\mathcal {J}({D})=\mathcal {J}'({D})$ for every ${D} \in \mathcal {U}$.

#### Proof

If ${D}\in \mathcal {U}$ and ${X_D^\theta }(0) = \left( {f_1N_0,\ldots , f_nN_0} \right) \in \Delta ^{\theta }$ for every $\theta \in \Theta$, then Lemma 2.1 implies that ${X_D^\theta (t)} \in \Delta ^{\theta } {\subset \Delta }$ for every ${t}\in [0,T]$. Recalling that, for every $\theta \in \Theta$, $F_0^\theta \equiv \tilde{F}_0^\theta$ and $F_1^\theta \equiv \tilde{F}_1^\theta$ on $B_{2{K_{\max }}}(0)\supset \Delta$ (see eq. (2.7)), it turns out that $\mathcal {J}({D})=\mathcal {J}'({D})$. $\square$

In view of Lemma 2.3, we now address the computation of the differential of $\mathcal {J}'$. The fact that $\mathcal {J}'$ is defined on the whole $L^2$ simplifies the arguments. Given ${D} \in L^2([0,T],\mathbb {R})$, we compute the differential of the functional $\mathcal {J}'$, and we represent it as an element of $L^2([0,T],\mathbb {R})$ via the Riesz’s isometry. Taking advantage of the results obtained in Scagliotti (2023a), we first consider the mapping $L^2([0,T],\mathbb {R}) \ni {D}\mapsto {X_D^\theta }(t) \in \mathbb {R}^n$ when ${t} \in [0,T]$ and $\theta \in \Theta$ are fixed.

#### Lemma 2.4

Let us consider ${D,\delta } \in L^2([0,T],\mathbb {R})$ and $\varepsilon \in (0,1]$. Let us denote with ${X_D^\theta }, {X_{D+\varepsilon \delta }^\theta }$ the solutions of eq. (2.13) corresponding, respectively, to the controls ${D, D+ \varepsilon \delta }$. Then, we have that

2.14 $$\begin{aligned} \sup _{{t}\in [0,T]} \sup _{\theta \in \Theta } | {X_{D+\varepsilon \delta }}^\theta ({t}) - {X_D^\theta } ({t}) - \varepsilon {Y_{D, \delta }^\theta } ({t}) | = o(\varepsilon ) \quad \text{ as } \varepsilon \rightarrow 0, \end{aligned}$$

where, for every ${t}\in [0,T]$ and for every $\theta \in \Theta$, we set

2.15 $$\begin{aligned} {Y_{D, \delta }^\theta } ({t}) :=M_{D}^\theta ({t}) \int _0^{t} \left( M_{D}^{\theta }(\sigma ) \right) ^{-1} F_1^\theta \left( {X_D^\theta }(\sigma ) \right) {\delta }(\sigma ) \,\textrm{d}\sigma , \end{aligned}$$

and $${t}\mapsto M_{D}^\theta ({t})\in \mathbb {R}^{{n}\times {n}}$$ solves

$$\begin{aligned} {\left\{ \begin{array}{ll} \dot{M}_{D}^\theta (\sigma ) = \left( \frac{\partial F_0^\theta ({X_D^\theta }(\sigma ))}{\partial x} + \frac{\partial F_1^\theta ({X_D^\theta }(\sigma ))}{\partial x} {D}(\sigma ) \right) M_{D}^\theta (\sigma ), \\ M_{D}^\theta (0) = \textrm{Id}. \end{array}\right. } \end{aligned}$$

#### Proof

See Appendix A. $$\square $$

We are now ready for providing a representation of the differential of the functional $\mathcal {J}'$. Here, we use $(\mathbb {R}^n)^*$ to denote the space of row-vectors, i.e., dual space of $\mathbb {R}^n$.

#### Proposition 2.5

Let ${\mathcal {J}':L^2([0,T],\mathbb {R})\rightarrow \mathbb {R}}$ be defined as in eq. (2.12), and let us consider ${D,\delta }\in L^2([0,T],\mathbb {R})$ and $\varepsilon \in [0,1]$. Then, the functional $\mathcal {J}'$ is Gateaux-differentiable at *D*, and we have that

2.16 $$\begin{aligned} \lim _{\varepsilon \rightarrow 0} \frac{\mathcal {J}'({D}+\varepsilon {\delta })- \mathcal {J}'({D})}{\varepsilon } = \int _0^T \left( \int _\Theta {g_D^\theta }(\sigma ) F_1^\theta \left( {X_D^\theta }(\sigma ) \right) \,\textrm{d}\mu (\theta ) \right) {\delta }(\sigma ) \,\textrm{d}\sigma , \end{aligned}$$

where ${g_D^\theta }:[0,T]\rightarrow (\mathbb {R}^{n})^*$ is an absolutely continuous curve that solves

2.17 $$\begin{aligned} {\left\{ \begin{array}{ll} {\dot{g}_D^\theta } (\sigma ) = -\ell ' \left( {N_D^\theta }(\sigma ) \right) (1,{\ldots ,}1) -{g_D^\theta }(\sigma ) \left( \frac{\partial F_0^\theta ({X_D^\theta }(\sigma ))}{\partial x} + \frac{\partial F_1^\theta ({X_D^\theta }(\sigma ))}{\partial x} {D}(\sigma ) \right) ,\\ {g_D^\theta } (T) = (0,{\ldots ,}0), \end{array}\right. } \end{aligned}$$

for every $$\theta \in \Theta $$.

#### Proof

Recalling that by construction the vector fields $F_0^\theta , F_1^\theta$ vanishes outside the set $B_{3K_{\max }}(0)\subset \mathbb {R}^{n}$ for every $\theta \in \Theta$, we deduce that there exists $\kappa >0$ such that ${N_{D+\varepsilon \delta }^\theta } ({t}) = (1,{\ldots ,}1)\cdot {X_{D+\varepsilon \delta }^\theta } \in [-\kappa ,\kappa ]$ for every $\theta \in \Theta$ and for every $\varepsilon \in [0,1]$. Recalling that $\ell :\mathbb {R}\rightarrow \mathbb {R}_+$ is of class $C^2$, owing to Lemma 2.4 we obtain that

$$\begin{aligned} \sup _{{t}\in [0,T]}\sup _{\theta \in \Theta } \left| \ell \left( {N_{D+\varepsilon \delta }^\theta }({t})\right) - \ell \left( {N_D^\theta }({t})\right) - \varepsilon \, \ell ' \left( {N_D^\theta }({t}) \right) (1,{\ldots ,}1)\cdot {Y_{D,\delta }^\theta }({t}) \right| = o(\varepsilon ) \quad \text{ as } \varepsilon \rightarrow 0. \end{aligned}$$

The last identity yields

2.18 $$\begin{aligned} \mathcal {J}'({D}+\varepsilon {\delta }) = \mathcal {J}'({D}) + \varepsilon \int _0^T \int _\Theta \ell ' \left( {N_D^\theta }({t}) \right) (1,{\ldots ,}1)\cdot {Y_{D,\delta }^\theta }({t}) \,\textrm{d}\mu (\theta ) \,\textrm{d}{t} + o(\varepsilon ) \quad \end{aligned}$$

as $\varepsilon \rightarrow 0$. We now focus on the first order term in eq. (2.18), and, taking advantage of eq. (2.15), we compute:

2.19 $$\begin{aligned} {\begin{matrix} & \int _0^T \int _\Theta \ell ' \left( {N_D^\theta }({t}) \right) (1,{\ldots ,}1) \cdot {Y_{D,\delta }^\theta }({t}) \,\textrm{d}\mu (\theta ) \,\textrm{d}{t} \\ & = \int _0^T \int _\Theta \ell ' \left( {N_D^\theta }({t}) \right) (1,{\ldots ,}1)\cdot M_{D}^\theta ({t}) \left[ \int _0^{t} \left( M_{D}^{\theta }(\sigma ) \right) ^{-1} F_1^\theta \left( {X_D^\theta }(\sigma ) \right) {\delta }(\sigma ) \,\textrm{d}\sigma \right] \,\textrm{d}\mu (\theta ) \,\textrm{d}{t} \\ & = \int _0^T \left[ \int _\Theta \int _\sigma ^T \ell ' \left( {N_D^\theta }({t}) \right) (1,{\ldots ,}1)\cdot M_{D}^\theta ({t}) \,\textrm{d}{t} \left( M_{D}^{\theta }(\sigma ) \right) ^{-1} F_1^\theta \left( {X_D^\theta }(\sigma ) \right) \,\textrm{d}\mu (\theta ) \right] {\delta }(\sigma ) \,\textrm{d}\sigma , \end{matrix}} \end{aligned}$$

where we used Fubini’s Theorem in the second identity. For every $\theta \in \Theta$ and for every $\sigma \in [0,T]$, we define

2.20 $$\begin{aligned} {g_D^\theta }(\sigma ) :=\int _\sigma ^T \ell ' \left( {N_D^\theta }({t}) \right) (1,{\ldots ,}1)\cdot M_{D}^\theta ({t}) \,\textrm{d}{t} \left( M_{D}^{\theta }(\sigma ) \right) ^{-1}, \end{aligned}$$

and, by combining eq. (2.18) to (2.20), we deduce that eq. (2.16) holds. We are left to show that ${g_D^\theta } :[0,T]\rightarrow (\mathbb {R}^{n})^*$ solves eq. (2.17). When $\sigma =T$, we directly read from eq. (2.20) that the terminal condition ${g_D^\theta }(T)=(0,{\ldots ,}0)$ is satisfied. By differentiating with respect to $\sigma$ the righ-hand side of eq. (2.20), we get

2.21 $$\begin{aligned} \dot{g_D^\theta }(\sigma ) = -\ell ' \left( {N_D^\theta }(\sigma ) \right) (1,{\ldots ,}1) + \int _\sigma ^T \ell ' \left( {N_D^\theta }({t}) \right) (1,{\ldots ,}1)\cdot M_{D}^\theta ({t}) \,\textrm{d}{t} \frac{\textrm{d}}{\textrm{d}\sigma } \left( M_{D}^{\theta }(\sigma ) \right) ^{-1}. \end{aligned}$$

Leveraging a classical result (see, e.g., Bressan and Piccoli 2007, Theorem 2.2.3), we obtain that

$$\begin{aligned} \frac{\textrm{d}}{\textrm{d}\sigma } \left( M_{D}^{\theta }(\sigma ) \right) ^{-1} = -\left( M_{D}^{\theta }(\sigma ) \right) ^{-1} \left( \frac{\partial F_0^\theta ({X_D^\theta }(\sigma ))}{\partial x} + \frac{\partial F_1^\theta ({X_D^\theta }(\sigma ))}{\partial x} {D}(\sigma ) \right) , \end{aligned}$$

and finally, combining the last identity with eq. (2.20) and (2.21), we finish the proof. $\square$

Proposition 2.5 yields the following result for the differential of the functional $\mathcal {J}$ related to the model of interest.

#### Corollary 2.6

Let $\mathcal {J}:\mathcal {U}\rightarrow \mathbb {R}$ be defined as in eq. (2.8), and let us consider ${D,D'}\in \mathcal {U}$ and $\varepsilon \in [0,1]$. Then, we have

2.22 $$\begin{aligned} \lim _{\varepsilon \rightarrow 0} \frac{\mathcal {J}({D+\varepsilon (D'-D)})- \mathcal {J}({D})}{\varepsilon } = \int _0^T \left( \int _\Theta {g_D^\theta }(\sigma ) F_1^\theta \left( {X_D^\theta }(\sigma ) \right) \,\textrm{d}\mu (\theta ) \right) {(D'(\sigma )-D(\sigma ))} \,\textrm{d}\sigma , \end{aligned}$$

where ${g_D^\theta } :[0,T]\rightarrow (\mathbb {R}^{n})^*$ solves eq. (2.17) for every $\theta \in \Theta$.

#### Proof

Recalling that $\mathcal {U}\subset L^2([0,T],\mathbb {R})$ is convex, we have that ${D+\varepsilon (D'-D)}\in \mathcal {U}$ for every $\varepsilon \in [0,1]$. Hence, Lemma 2.3 guarantees that $\mathcal {J}({D+\varepsilon (D'-D)})=\mathcal {J}'({D+\varepsilon (D'-D)})$ for every $\varepsilon \in [0,1]$. Therefore, we conclude using Proposition 2.5. $\square$

From Corollary 2.6 we readily get the representation of the differential of $\mathcal {J}$. Namely, for every ${D}\in \mathcal {U}$, owing to eq. (2.22) we can represent through Riesz’s isometry the differential $\nabla _{D}\mathcal {J}$ as

2.23 $$\begin{aligned} \nabla _{D} \mathcal {J}({t}) :=\int _\Theta {g_D^\theta }({t}) F_1^\theta \left( {X_D^\theta }({t}) \right) \,\textrm{d}\mu (\theta ) \end{aligned}$$

for every ${t} \in [0,T]$. We conclude this part by observing the structure of a gradient-based algorithm for the minimization of $\mathcal {J}$. We first investigate the tangent cone to the set of admissible control $\mathcal {U}$ at a point ${D}\in \mathcal {U}$. In doing this, we follow the notion provided in (Mordukhovich 2006, Definition 1.8), i.e.,

2.24 $$\begin{aligned} T({D},\mathcal {U}) :=\limsup _{\varepsilon \rightarrow 0}\frac{\mathcal {U}- {D}}{\varepsilon }, \end{aligned}$$

where the $\limsup$ in eq. (2.24) is understood as the collection of the $L^2$-strong limiting points of sequences $(v_{\varepsilon _n})_n$ such that $v_{\varepsilon _n}\in \frac{\mathcal {U}- {D}}{\varepsilon _n}$ and $\varepsilon _n\rightarrow 0$ as $n\rightarrow \infty$.

#### Lemma 2.7

Let $\mathcal {U}\subset L^2([0,T],\mathbb {R})$ be defined as in eq. (2.6). Then, for every ${D}\in \mathcal {U}$, we have that

2.25 $$\begin{aligned} T({D},\mathcal {U}) = \left\{ v \in L^2([0,T],\mathbb {R}) : v({t}) \le 0 \,\,\, \textrm{ if }\,\,\, {D}({t})={D_{\max }},\, v(\tau ) \ge 0 \,\,\, \textrm{ if }\,\,\, {D}({t})=0 \right\} . \end{aligned}$$

#### Proof

See Appendix A. $$\square $$

We introduce the mappings $\Pi _\mathcal {U}:L^2([0,T],\mathbb {R}) \rightarrow \mathcal {U}$ and $\Pi _{T({D},\mathcal {U})} :L^2([0,T],\mathbb {R}) \rightarrow T({D},\mathcal {U})$ defined as the projections onto the closed convex set $\mathcal {U}$ and onto the closed convex cone $T({D},\mathcal {U})$, respectively. With a direct computation, it is possible to show that

2.26 $$\begin{aligned} \Pi _\mathcal {U}[v]({t}) = \max \big (\min \big ( v({t}), {D_{\max }}\big ), 0\big ) \end{aligned}$$

and that

2.27 $$\begin{aligned} \Pi _{T({D},\mathcal {U})}[v]({t}) = \max \big ( v({t}), 0 \big ) 1\!\!1_{\{{D}=0\}} + \min \big ( v({t}), 0 \big ) 1\!\!1_{\{{D}={D_{\max }}\}} + v({t})1\!\!1_{\{0<{D}<{D_{\max }}\}} \end{aligned}$$

for every ${t}\in [0,T]$, for every ${D}\in \mathcal {U}$ and for every $v\in L^2([0,T],\mathbb {R})$.

#### Proposition 2.8

Let $\mathcal {J}:\mathcal {U}\rightarrow \mathbb {R}$ be defined as in eq. (2.8), and let us consider $\eta >0$. Then, for every ${D}\in \mathcal {U}$ we have that

$$\begin{aligned} \Pi _\mathcal {U}\left[ {D} - \eta \nabla _{D} \mathcal {J}\right] = \Pi _\mathcal {U}\left[ {D} + \eta \Pi _{T({D},\mathcal {U})} [-\nabla _{D} \mathcal {J}] \right] . \end{aligned}$$

#### Proof

To ease the notations, let us define $v_1 :={D} - \eta \nabla _{D} \mathcal {J}$ and $v_2 :={D} + \eta \Pi _{T({D},\mathcal {U})} [-\nabla _{D} \mathcal {J}]$. From eq. (2.26) and (2.27), we notice that $-\nabla _{D} \mathcal {J}$ can differ from $\Pi _{T({D},\mathcal {U})} [-\nabla _{D} \mathcal {J}]$ only on those points ${t}\in [0,T]$ such that either ${D}({t})=0$ or ${D}({t})={D_{\max }}$.

Let us assume that for some ${t}\in [0,T]$ we have ${D}({t})=0$. On the one hand, if $-\nabla _{D} \mathcal {J}({t})>0$, then $-\nabla _{D} \mathcal {J}({t}) = \Pi _{T({D},\mathcal {U})} [-\nabla _{D} \mathcal {J}]$, yielding $v_1({t})=v_2({t})$ and $\Pi _\mathcal {U}[v_1]({t})= \Pi _\mathcal {U}[v_2]({t})$. On the other hand, if $-\nabla _{D} \mathcal {J}({t})<0$, then $\Pi _{T({D},\mathcal {U})} [-\nabla _{D} \mathcal {J}]=0$, so that $v_2({t})=0$, while $v_1({t})<0$. However, using eq. (2.26), we deduce that $\Pi _\mathcal {U}[v_1]({t})= 0 = \Pi _\mathcal {U}[v_2]({t})$.

The argument for *t* such that ${D}({t})={D_{\max }}$ is analogous. $\square$

#### Remark 5

The previous result suggests an implementable approach for the numerical minimization of the functional $\mathcal {J}$. Given a current guess ${D}_n\in \mathcal {U}$, we perform the update ${D}_{n+1}:=\Pi _{\mathcal {U}}[{D}_n - \eta \nabla _{{D}_n}\mathcal {J}]$. Proposition 2.8 ensures that we do not need to take care of projecting $-\nabla _{{D}_n}\mathcal {J}$ onto the tangent cone to $\mathcal {U}$ at ${D}_n$. The convergence properties of the proposed projected gradient scheme (in particular, convergence to stationary points) follow from classical results on projected gradient methods in Hilbert spaces; see (Bertsekas 1999, Chapter 2).

## Ensemble Optimal Control in Action: Numerical Experiments

In this section, we first describe the framework where we set our numerical experiments^2^, i.e., the androgen deprivation therapy in prostate cancer. Then, we present the benchmark strategies and we discuss the results obtained through the resolution of ensemble optimal control problems. Finally, taking advantage of the insights provided by this viewpoint, we introduce a variant of the adaptive therapy proposed in Gatenby et al. (2009); Zhang et al. (2017).

In the remainder of this paper, we work within the two-population model mentioned in the Introduction (see eq. (1.5) and Table 2). As usual, in order to get a non-dimensional system of equations, we perform in eq. (1.5) the time reparametrization and the function transformations as follows:

3.1 $$\begin{aligned} {\begin{matrix} & \tau :=r_S t, \quad s(\tau ) :=\frac{S(r_S t)}{K}, \quad r(\tau ) :=\frac{R(r_S t)}{K},\\ & n(\tau ) :=\frac{N(r_S t)}{K}, \quad u(\tau ) :=\frac{D(r_S t)}{D_{\max }}. \end{matrix}} \end{aligned}$$

We observe that in eq. (1.5) $r_S$ denotes the reproduction rate of the sensitive cells, and it is employed for the change in the time-scale. Consequently, when using the time variable $\tau$, the sensitive cells have unitary reproduction rate. Finally, we rescale as well the time-independent parameters appearing in eq. (1.5):

3.2 $$\begin{aligned} \hat{d}_D :=2 d_D, \quad \hat{d}_T :=\frac{d_T}{r_S}, \quad \hat{r}_R :=\frac{r_R}{r_S}. \end{aligned}$$

We collect in $\theta :=(\hat{d}_D, \hat{d}_T, \hat{r}_R, \hat{f}_0)$ the non-negative constants that parametrize the ensemble of systems described below, and that affect both the dynamics and the Cauchy datum. Therefore, we obtain the following non-dimensional equations:

3.3 $$\begin{aligned} {\begin{matrix} & {\left\{ \begin{array}{ll} \dot{s}^\theta _u(\tau ) = \big ( 1 - s^\theta _u(\tau ) - r^\theta _u(\tau ) \big ) \left( 1 - \hat{d}_D u(\tau ) \right) s^\theta _u(\tau ) - \hat{d}_T s^\theta _u(\tau ), \\ \dot{r}^\theta _u(\tau ) = \hat{r}_R \big ( 1 - s^\theta _u(\tau ) - r^\theta _u(\tau ) \big ) r^\theta _u(\tau ) - \hat{d}_T r^\theta _u(\tau ), \end{array}\right. } \\ & \quad n^\theta _u(\tau ) :=s^\theta _u(\tau ) + r^\theta _u(\tau ), \end{matrix}} \end{aligned}$$

where $\tau \mapsto u(\tau ) \in [0,1]$ is the control which we act on the system with, and $\tau \mapsto s^\theta _u(\tau )$, $\tau \mapsto r^\theta _u(\tau )$ are the corresponding trajectories (see Table 3). We denote with $n_0\in (0,1)$ the initial tumor size (i.e., $n^\theta (0)=n_0$), and we set the Cauchy datum for (3.3) to be

3.4 $$\begin{aligned} r^\theta _u(0) = r_0^\theta = \hat{f}_0n_0, \qquad s^\theta _u(0) = s_0^\theta =(1-\hat{f}_0)n_0, \end{aligned}$$

with $$\hat{f}_0 \in [0,1]$$.

**Table 3:** Non-dimensional variables and parameters of the simplified model.

**Parameter**	**Meaning**
$s(\tau )$	Normalized size of the sensitive subpopulation
$r(\tau )$	Normalized size of the resistant subpopulation
$n(\tau )$	Total normalized population $s(\tau )+r(\tau )$
$\tau$	Rescaled (dimensionless) time
$u(\tau )$	Normalized drug dosage (control)
$\hat{d}_D$	Rescaled drug sensitivity coefficient
$\hat{d}_T$	Rescaled turnover (death) rate
$\hat{r}_R$	Relative proliferation rate of resistant cells (with respect to sensitive cells)
$\hat{f}_0$	Initial fraction of resistant cells
$n_0$	Initial total normalized population

We insist on the fact that eq. (3.3) does not include a mechanism of secondary (or ‘acquired’) resistance, and the population $r^\theta (\tau )$ at $\tau >0$ is made of descendants of the resistant clones $r_0^\theta$ present from the very beginning. This hypothesis is frequently assumed (see, e.g., Cunningham et al. 2018; Strobl et al. 2021) and is consistent with the bio-medical evidences concerning the heterogeneity of cancer cells (Swanton 2012).

### Framework and Parameters Setting

In this part, we consider the parameters appearing in eq. (3.3) and (3.4) and define the ranges where they vary. In doing that, we take advantage of the estimates provided in Strobl et al. (2021) for *prostate cancer treated with androgen deprivation therapy (ADT)*. We recall that the analysis in Strobl et al. (2021) relied on the results of the trial described in Bruchovsky et al. (2006). There, a cohort of patients with biochemical recurrence of the tumor (non-metastatic and castration sensitive, m0CSPC) after radical radiotherapy treatment underwent intermittent ADT.

#### Parameters not affected by uncertainty

We begin by listing the parameters that in our experiments are assumed to be unaffected by uncertainty. This restriction is motivated by the fact that some parameters are taken from well-established estimates available in the literature. Furthermore, limiting the number of uncertain parameters allows us to study the variability in treatment response, while keeping the ensemble optimal control problem computationally tractable.

The first crucial quantity to be estimated is the proliferation rate for sensitive cells $r_S$, which is employed to perform the time-normalization of the original system (1.5) into (3.3). In Strobl et al. (2021), the authors set $r_S=0.027\, \textrm{day}^{-1}$, adopted from a previous estimate derived in Zhang et al. (2017). Then, the effectiveness of the therapy is encoded in the value of the non-dimensional constant $\hat{d}_D$, which is set $\hat{d}_D=1.5$ in Strobl et al. (2021) (see also West et al. 2019 for the original estimate). We recall that the normalized dimension of the tumor is expressed using $n=n(\tau )$, which ranges in [0, 1]. Here, $n=0$ means that the tumor cells (sensitive and resistant) are entirely extinct, while $n=1$ implies that the tumor size has reached the system’s carrying capacity. The initial size of the tumor $n(0)=n_0$ in our experiments takes values in $\{0.25, 0.50, 0.75\}\ni n_0$. We insist that $n_0$ is not affected by uncertainty, and we shall run the simulations separately for the different values of $n_0$. Finally, in Strobl et al. (2021) the turnover rate $\hat{d}_T$ is proposed to range in the interval $\hat{d}_T\in [0,0.5]$. In the present paper, we consider the most adverse scenario, i.e., we always set $\hat{d}_T=0$.

#### Parameters affected by uncertainty

We now describe the parameters that are affected by uncertainty. The first quantity is the normalized proliferation rate of the resistant tumor cells encoded in $\hat{r}_R$. In Strobl et al. (2021) this constant is suggested to vary in the interval $[0.5,1]\ni \hat{r}_R$. Here, $\hat{r}_R= 0.5$ means that the ‘cost’ of having the drug resistance results in the proliferation rate being $50\%$ smaller than that of sensitive cells. Conversely, if $\hat{r}_R= 1$, the resistant cells have no disadvantage towards the sensitive ones regarding reproduction rate.

In the ODE model considered here, there is no mechanism of resistance acquirement during the dynamics. In other words, in our framework, the population of resistant cells $r(\tau )$ at an instant $\tau >0$ consists of clones of an initial resistant subpopulation that is assumed to be present from the very beginning. This hypothesis is consistent with the heterogeneity and polyclonal nature of solid cancers, which in the clinical practice is particularly realistic in the case of advanced or metastatic disease (see e.g. Swanton 2012). We assume that the initial fraction $\hat{f}_0$ of resistant cells ranges in $[0.002,0.1]\ni \hat{f}_0$, meaning that the ratio $\hat{f}_0:=r(0)/(r(0)+s(0))$ varies between $0.2\%$ (most favourable scenario) and $10\%$ (worst-case scenario). In future work, we plan to incorporate a feature that accounts for the secondary resistance mechanism into the model.

#### Space of parameters and ensemble measure

We are in a position to provide a formal definition for the space of parameters $\Theta$ where $\theta :=(\hat{d}_D, \hat{d}_T, \hat{r}_R, \hat{f}_0)$ takes values. According to what was said above, in our experiments we consider

$$\begin{aligned} \Theta = \{1.5\} \times \{0\} \times [0.5,1] \times [0.002,0.1]. \end{aligned}$$

In the current preliminary work, we assume the uncertain parameters $\hat{r}_R, \hat{f}_0$ to be *independent* and *uniformly distributed* in the respective intervals where they range. This hypothesis implies that the probability measure $\mu \in \mathcal {P}(\Theta )$ used for defining the ensemble optimal control problem has the form:

3.5 $$\begin{aligned} \mu :=\delta _{1.5} \otimes \delta _0 \otimes U(0.5,1) \otimes U(0.002,0.1), \end{aligned}$$

where $\delta _{z}$ denotes the Dirac delta centered at $z\in \mathbb {R}$, and *U*(*a*, *b*) denotes the uniform probability distribution over the interval [*a*, *b*].

##### Remark 6

The theoretical machinery developed in Section 2 is completely general and does not require the independence of the different parameters that appear in the model. If, in the future, evidence of correlations between such parameters is observed, it will suffice to adapt accordingly the choice of the measure $\mu$. Similarly, if we wished to consider random fluctuations in $\hat{d}_D$ or $\hat{d}_T$, we could right away include these features in $\Theta$ and $\mu$.

As observed in Remark 3, any ensemble optimal control problem related to the measure $\mu$ defined as in eq. (3.5) requires the *simultaneous* solution of infinitely many ODEs, just for evaluating the objective functional. Since this is unfeasible for simulations, we pursue the approach provided by Theorem 2.2. Namely, we approximate $\mu$ with a discrete probability measure $\mu _k$ with finite support, and we address the minimization of the functional related to $\mu _k$. We emphasize that the construction of $\mu _k$ detailed below should be understood as a proof of concept. In a more realistic scenario, one can consider repeated measurements of tumor burden in a cohort of *k* patients and estimate the model parameters using techniques from system identification (see, e.g., Nelles 2020). The model parameters for each patient, $\theta _1,\ldots ,\theta _k$, are then interpreted as independent samples from the measure $\mu$, and the resulting empirical probability measure $\mu _k :=\frac{1}{k} \sum _{i=1}^k \delta _{\theta _i}$ is used in the definition of the functional $\mathcal {J}_k$. A detailed description of such parameter estimation procedures is beyond the scope of the present manuscript and is left for future development. Our focus here is on the mathematical formulation and analysis of the ensemble optimal control problem, rather than on the practical implementation of parameter estimation.

In our specific setting, we discretized the interval [0.5, 1] where $\hat{r}_R$ varies into 25 equispaced nodes with distance 0.02. Hence, we set $U(0.5,1)\approx \frac{1}{25}\sum _{j=1}^{25} \delta _{\hat{r}_R^j}$, with $\hat{r}_R^j\in \{0.5, 0.52,\ldots ,1\}$. Regarding the parameter $\hat{f}_0$, we divided the interval [0.002, 1] into 49 nodes with a constant step equal to 0.002. Then, we considered $U(0.002,0.1)\approx \frac{1}{49}\sum _{k=1}^{49} \delta _{\hat{f}_0^k}$, with $\hat{f}_0^k\in \{0.002, 0.004,\ldots ,0.1\}$. Finally, we defined

3.6 $$\begin{aligned} \Theta _N :=\{1.5\} \times \{0\} \times \{0.5,0.52,\ldots ,1\} \times \{0.002,0.004, \ldots , 0.1\}, \end{aligned}$$

and

3.7 $$\begin{aligned} \mu _N:=\delta _{1.5} \otimes \delta _0 \otimes \frac{1}{25}\sum _{j=1}^{25} \delta _{\hat{r}_R^j} \otimes \frac{1}{49}\sum _{k=1}^{49} \delta _{\hat{f}_0^k}, \end{aligned}$$

which has a support consisting of $N=1225$ points. From a practical perspective, the introduction of $\mu _N$ results in performing the numerical experiments on $N=1225$ different tumors, corresponding to $\theta ^{j,k}=(1.5,0,\hat{r}_R^j, \hat{f}_0^k)$ with $j=1,\ldots ,25$ and $k=1,\ldots ,49$. We used these simulated tumors to evaluate every therapy schedule that we considered.

#### Numerical integration scheme

For every treatment schedule that we tested, we approximated the dynamics of the control systems (3.3) using the Explicit Euler method with stepsize $\tau _{\textrm{discr}}=r_S/8=3.375\cdot 10^{-3}$. In the non-rescaled system (1.5), this choice corresponds to dividing each simulation day into 8 equispaced time nodes.

#### Time-To-Progression (TTP)

In this paper, we measure the performances of the different strategies of drug scheduling using the Time-To-Progression (TTP), i.e., the length of the period from $\tau =0$ to the first instant $\tau _{\textrm{TTP}}>0$ when the tumor is $20\%$ bigger (in terms of population) than the initial size $n_0$. The disease is said ‘to be in progression’ when this condition occurs. More precisely, for every $\theta \in \Theta _N$ we set

3.8 $$\begin{aligned} \tau _{\textrm{TTP}}^\theta :=\inf _{\tau >0}\{ s^\theta (\tau ) + r^\theta (\tau ) \ge 1.2\, n_0 \}. \end{aligned}$$

We report that this quantity was employed also in Strobl et al. (2021), and we recall that is related to the radiologically-defined criterion ‘RECIST’ (see Eisenhauer et al. 2009). In view of the strategies resulting from ensemble optimal control problems, it is convenient to introduce a variant of TTP, which we denote with $\hbox {TTP}^\prime$. We define it as follows:

3.9 $$\begin{aligned} \tau _{\mathrm {TTP'}}^\theta :=\sup _{\tau >0}\{ s^\theta (\tau ) + r^\theta (\tau ) \le 1.2\, n_0 \} \end{aligned}$$

for every $$\theta \in \Theta _N$$.

##### Remark 7

Since $s^\theta (0) + r^\theta (0)=n_0< 1.2 \, n_0$, the continuity of the mapping $\tau \mapsto s^\theta (\tau ) + r^\theta (\tau )$ for every $\theta$ implies that $\tau _{\textrm{TTP}}^\theta \le \tau _{\mathrm {TTP'}}^\theta$. If for some $\theta$ there exists a unique instant $\bar{\tau }>0$ such that $s^\theta (\bar{\tau }) + r^\theta (\bar{\tau }) = 1.2\, n_0$, then it turns out that $\tau _{\textrm{TTP}}^\theta = \tau _{\mathrm {TTP'}}^\theta = \bar{\tau }$.

### Benchmark Approaches: MTD and Adaptive Therapy

We begin by describing the benchmark therapies for evaluating the performances of the schedules proposed later. The baseline is the Maximal Tolerated Dose protocol (MTD), which consists of constantly giving the patient the maximal quantity of the drug. In our model, this results in setting $u_{\textrm{MTD}}(\tau ) = 1$ for every $\tau >0$. When the competition between sensitive and resistant clones for shared resources is relevant (like in the present model), MTD has already been shown to be sub-optimal. For instance, the role played by this Darwinian mechanism was highlighted in Silva et al. (2012). Namely, the MTD leads rapidly to the extinction of the sensitive population, resulting in a quick and dramatic shrinking of the tumor size. After observing partial or total regression of the disease for a certain amount of time—whose duration depends indeed on the proliferation rate $\hat{r}_R$ of the resistant cells—, a resurgence of the tumor is typically observed, and the resistant cells are predominant.

Adaptive Therapy (AT) has been introduced in Gatenby et al. (2009) for enhancing the long-time management of the disease, and it has been proven effective in several studies involving both numerical simulations (Zhang et al. 2017) and a pilot study with patients (Zhang et al. 2017, 2022). The key idea is to adaptively introduce *vacation periods* when drug treatment is discontinued. Here, we apply the strategy as described in Strobl et al. (2021) on the ensemble of tumors modeled by $\Theta _N$ (see eq. (3.6)) and which we simulate the dynamics of. More precisely, the AT relies on the following steps, for every $\theta \in \Theta _N$:

1. At $\tau =0$, set $u=1$ and keep it constant while $s^\theta (\tau ) + r^\theta (\tau )> n_0/2$ (treatment period).
2. After observing $s^\theta (\tau )+r^\theta (\tau )\le n_0/2$, reset $u=0$ and keep it constant while $s^\theta (\tau ) + r^\theta (\tau )< n_0$ (vacation period).
3. After observing again $s^\theta (\tau ) + r^\theta (\tau ) \ge n_0$, reset $u=1$ and go to Step (1).

To distinguish between this approach and the variant of AT that we will discuss later, we refer to the strategy just described as ‘On-Off’ AT. The results are reported in Table 4. We remark that in the experiments involving MTD and ‘On-Off’ AT we observed $\tau _{\textrm{TTP}}^\theta = \tau _{\mathrm {TTP'}}^\theta$ for every $\theta \in \Theta _N$ (see Remark 7, and eq. (3.8) and (3.9) for the definitions).

**Table 4:** MTD and ‘On-Off’ AT.

	$n_0$	max TTP	min TTP	mean TTP
MTD	0.25	521 days	109 days	210 days
‘On-Off’ AT	0.25	575 days	109 days	213 days
MTD	0.50	593 days	155 days	270 days
‘On-Off’ AT	0.50	764 days	155 days	285 days
MTD	0.75	748 days	283 days	410 days
‘On-Off’ AT	0.75	1196 days	283 days	462 days

Comparison in terms of Time-to-Progression (TTP) between MTD and ‘On-Off’ AT. The mean TTP is computed by taking the average over the elements of the set $\Theta _N$ (see eq. (3.6))

**Fig. 1:**
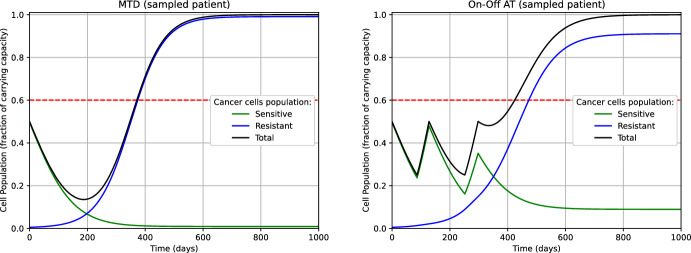
Dynamics of the tumor corresponding to $\theta = (1.5,0,0.66,0.01)$ and with initial size $n_0=0.5$ undergoing MTD (left) and ‘On-Off’ AT (right). The dashed horizontal line represents the threshold tumor size related to the condition ‘cancer in progression’. For this $\theta$, the advantage of ‘On-Off’ AT (right picture) over MTD (left picture) is apparent. The TTP of ‘On-Off’ AT and MTD is 424 days and 370 days, respectively, with a progression delay of almost 8 weeks (color figure online)

The values of the TTP in Table 4 are perfectly consistent with the findings shown in (Strobl et al. (2021), Figure 3.A). Namely, on the one hand, the ‘On-Off’ AT never performed worse than the classical MTD. On the other hand, the advantage of the introduction of ‘treatment vacation periods’ in AT is apparent when $n_0=0.5$ and $n_0=0.75$, where the progression of the disease is delayed respectively of 2 and 7 weeks on average, if compared to the mean TTP achieved using MTD. In fig. 1, we plotted the dynamics of the tumor populations (sensitive, resistant, and total) when treated with MTD and ‘On-Off’ AT, for a specific value of the parameter $\theta = (\hat{d}_D, \hat{d}_T, \hat{r}_R, \hat{f}_0)$. For such a value of $\theta$, ‘On-Off’ AT dramatically outperforms MTD. On the one hand, in MTD the sensitive subpopulation is rapidly extinct, and it is replaced by the resistant cells, which do not have any biological competitor for resources. On the other hand, in ‘On-Off’ AT the ‘treatment vacation periods’ allow for restocking the sensitive subpopulation, preventing its extinction. This results in an increased fight between sensitive and resistant cells for shared resources, contributing to the delay of the progression of the disease. Moreover, we notice that in ‘On-Off’ AT we can directly read the treatment-vacation cycles from the graph of the populations’ dynamics.

### Ensemble Optimal Control Problems: Formulation and Resolution

In this part, we consider the problem of finding a therapy schedule $u=u(\tau )$ for the control system (3.3), in the case some of the parameters appearing in (3.3) are affected by uncertainty. We insist that, when solving an ensemble optimal control problem, we aim to find a shared policy $u=u(\tau )$ that shall be used *for every admissible tuple of parameters*. In Subsection 3.1 we introduced the space of parameters and the discrete measure $\mu _N$. Before addressing the resolution of the ensemble optimal control problem, we are left to set the time horizon for the controlled dynamics and the function $\ell :\mathbb {R}\rightarrow \mathbb {R}$ that designs the integral cost.

***Simulation interval*** For every $n_0\in \{0.25,0.50,0.75\}$ we set the value of the time horizon $T_{\textrm{hor}}$ according to the maximal value of TTP observed in Table 4. We collect the selected values in Table 5, where we also reported the normalized time $T:=T_{\textrm{hor}} \cdot r_S$ that is required in the definition of the functional $\mathcal {J}$ (see eq. (2.8)). We recall that $r_S=0.027 \,\textrm{days}^{-1}$ denotes the proliferation rate of the sensitive cells, and it has been used for the time-normalization of the original system (1.5) into (3.3).

**Table 5:** Time horizon.

$n_0$	$T_{\textrm{hor}}$	*T*
0.25	750 days	20.25
0.50	1000 days	27.00
0.75	1500 days	40.50

Values of $T_{\textrm{hor}}$ used for the optimal control problems.

#### Remark 8

The choice of $T_{\textrm{hor}}$ in Table 5 may sound arbitrary, and to some extent, it is. For every $n_0$, we set $T_{\textrm{hor}}$ to be $\approx 30\%$ larger than the best TTP obtained through ‘On-Off’ AT. However, as we will discuss in detail later in Remark 9, the resolution of an ensemble optimal control problem tends automatically to find the interval where the decision-making on the therapy schedule has the most significant effect.

***Integral cost design*** A crucial step in resolving any (ensemble) optimal control problem is the definition of the objective functional to be minimized. In view of providing an explicit expression for the functional $\mathcal {J}:\mathcal {U}\rightarrow \mathbb {R}$, we need to specify the function $\ell :\mathbb {R}\rightarrow \mathbb {R}$ involved in the integral cost. Here, we propose and compare two possible alternatives for $\ell$. The first natural attempt consists of setting

**Fig. 2:**
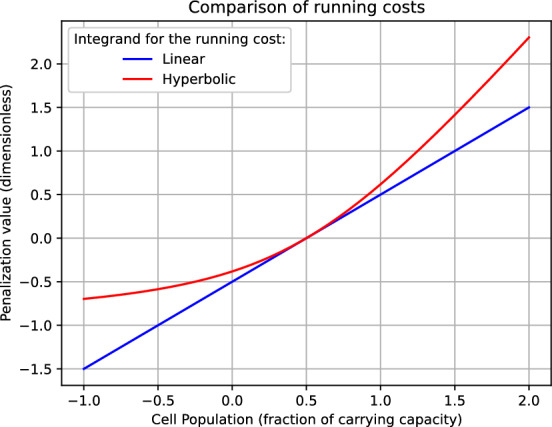
Graph of $\ell ^1$ (linear) and $\ell ^2$ (hyperbolic) for $n_0=0.50$ (see, respectively, eq. (3.10) and (3.11))

3.10 $$\begin{aligned} \ell ^1(n) :=n-n_0, \end{aligned}$$

which results in linearly penalizing any deviation of the tumor size $n^\theta (\tau ) = s^\theta (\tau ) + r^\theta (\tau )$ from the initial condition $n_0$. We observe that $\ell ^1$ has a symmetric behavior around $n_0$: If $\delta >0$ denotes a variation of the tumor size, a decrease amounting to $-\delta$ (i.e., $n=n_0-\delta$) is awarded a ‘negative cost’ $-\delta$, which in absolute value is as much as $\ell ^1$ penalizes the growth $n=n_0+\delta$. This property of $\ell ^1$ does not fully reflect the physicians’ aim when treating a patient for long-term disease control. Indeed, *the goal is to stabilize the tumor as long as possible rather than to eradicate it*. For this reason, we consider also the hyperbolic function $\ell ^2$ defined as follows:

3.11 $$\begin{aligned} \ell ^2(n) :=\sqrt{1 +(n-n_0)^2} -1 + (n-n_0), \end{aligned}$$

whose behavior around $n_0$ is not symmetric (see fig. 2). In this way, a positive deviation is penalized more than the corresponding negative deviation is awarded.

Nonetheless, the design of a proper integral cost for the long-term cancer management is an interesting and delicate problem that goes beyond the scope of the present paper. We leave open this point for future developments.

**Minimization of the cost functionals**

For every $n_0\in \{0.25,0.5,0.75\}$ we considered the functionals $\mathcal {J}^1,\mathcal {J}^2:\mathcal {U}\rightarrow \mathbb {R}$ defined as follows:

3.12 $$\begin{aligned} \mathcal {J}^1(u) :=\int _0^T \int _\Theta \ell ^1 \left( n_u^\theta (\tau )\right) \,\textrm{d}\mu _N(\theta ) \,\textrm{d}\tau , \qquad \mathcal {J}^2(u) :=\int _0^T \int _\Theta \ell ^2 \left( n_u^\theta (\tau )\right) \,\textrm{d}\mu _N(\theta ) \,\textrm{d}\tau , \end{aligned}$$

where $\ell ^1,\ell ^2$ have been introduced in eq. (3.10) and (3.11), *T* is set according to Table 5, and $\mu _N$ is the discrete measure on the ensemble of parameters that we defined in eq. (3.7). We used the following gradient-based minimization scheme for the numerical minimization of $\mathcal {J}^1$ and $\mathcal {J}^2$ (see Proposition 2.8):

3.13 $$\begin{aligned} u^i_{k+1} \leftarrow \Pi _\mathcal {U}\left[ u_k^i - \eta \nabla _{u_k^i} \mathcal {J}^i \right] \qquad k\ge 0, \quad i=1,2, \end{aligned}$$

where $\Pi _\mathcal {U}:L^2([0,T], \mathbb {R}) \rightarrow \mathcal {U}$ is the projection onto $\mathcal {U}$ (see eq. (2.26)), and where we set $\eta =0.125$. In order to avoid the introduction of any bias towards turning the therapy on or off, we considered as an initial guess the control $u^i_0\equiv 0.5$ for $i=1,2$, which is at every instant equidistant from 1 (full-dosage) and 0 (discontinued therapy). We repeated the step described in eq. (3.13) for 500 iterations. We implemented the simulations in Python, and we relied on the automatic differentiation tools of Pytorch. We also performed numerical simulations on the minimax problems related to the functionals functionals $\mathcal {J}^1_{\max },\mathcal {J}^2_{\max } :\mathcal {U}\rightarrow \mathbb {R}$ defined as follows:

$$\begin{aligned} \mathcal {J}^1_{\max } (u) :=\max _{\theta \in \Theta _N} \left\{ \int _0^T \ell ^1 \left( n_u^\theta (\tau )\right) \,\textrm{d}\tau \right\} , \quad \mathcal {J}^2_{\max } (u) :=\max _{\theta \in \Theta _N} \left\{ \int _0^T \ell ^2 \left( n_u^\theta (\tau )\right) \,\textrm{d}\tau \right\} . \end{aligned}$$

For this task, we employed the subgradient-like method proposed in (Scagliotti 2025, Algorithm 1).

#### Remark 9

**Fig. 3:**
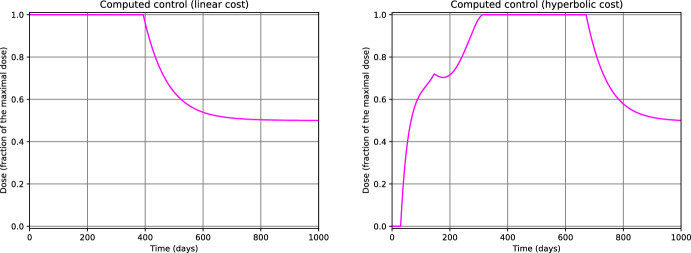
Controls computed by minimizing $\mathcal {J}^1$ (left) and $\mathcal {J}^2$ (right) for $n_0=0.50$. The profiles corresponding to $n_0=0.25$ and $n_0=0.75$ are qualitative similar. (color figure online)

**Fig. 4:**
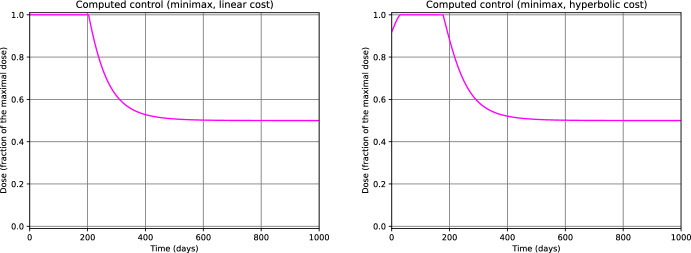
Controls computed by minimizing $\mathcal {J}^1_{\max }$ (left) and $\mathcal {J}^2_{\max }$ (right) for $n_0=0.50$. The profiles corresponding to $n_0=0.25$ and $n_0=0.75$ are qualitative similar. (color figure online)

From fig. 3 we notice that, for $\tau$ close to *T*, the computed controls $u^1_{500}$ and $u^2_{500}$ for $n_0=0.50$ do not deviate significantly from the value of the initial guess $u^1_{0}=u^2_0 \equiv 0.5$. We observed a similar behavior for $n_0=0.25$ and $n_0=0.75$ as well. This phenomenon is since, throughout the iterations of the gradient descent $k=0,\ldots ,500$, $\nabla _{u_k^1} \mathcal {J}^1(\tau )$ and $\nabla _{u_k^2} \mathcal {J}^2(\tau )$ are small when $\tau$ is close to *T*. From the model perspective, this suggests that, at later stages of the simulation horizon (i.e., when $\tau$ is close to *T*), the differences in the treatment strategy do not have a relevant impact on the final outcome. Intuitively, if for $\tau \ge \bar{\tau }$ most of the tumors of the ensemble $\Theta _N$ have already progressed to the carrying capacity of the system, then we expect that any change in the treatment policy after the instant $\bar{\tau }$ will not drastically improve the result. We can use this phenomenon as an empirical test for deciding whether the time horizon *T* has been appropriately set large (see Remark 8). Finally, we notice that the same phenomenon can be observed in the graph of the computed controls for $\mathcal {J}^1_{\max }$ and $\mathcal {J}^2_{\max }$ (Fig. 4).

**Results**

**Fig. 5:**
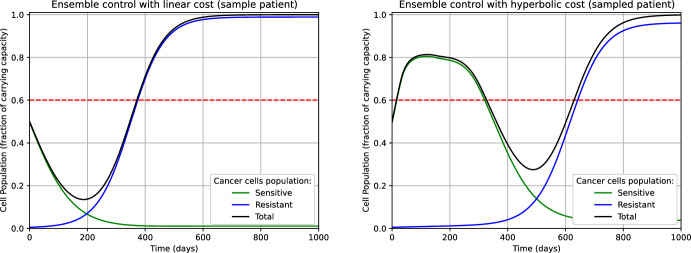
Dynamics of the tumor corresponding to $\theta = (1.5,0,0.66,0.01)$ and with initial size $n_0=0.5$ using the treatment prescribed by the approximated minimizers of $\mathcal {J}^1$ (linear cost, left) and of $\mathcal {J}^2$ (hyperbolic cost, right). The dashed horizontal line represents the threshold tumor size related to the condition ‘cancer in progression’. (color figure online)

We first show in fig. 3 the profiles of the controls computed via the gradient-based algorithm outlined above. Interestingly, the integral costs $\ell ^1$ and $\ell ^2$ tend to select optimal controls showing rather different qualitative behavior. Indeed, on the one hand, the computed optimal control for the functional $\mathcal {J}^1$—which incorporates the *linear* size penalization of the tumor—suggests giving the patient the maximal drug dose for a rather long initial interval. For instance, as we can read from fig. 3, at the end of the optimization procedure for $n_0=0.50$, $u^1_{500}$ is constantly equal to 1 in the first $\approx 400 \,\textrm{days}$. For this reason, when using this strategy, we expect to observe performances in terms of $\textrm{TTP}$ very close to the ones reported in Table 4 for MTD. On the other hand, the *hyperbolic* size penalization involved in the definition of $\mathcal {J}^2$ induces an entirely different profile in the computed control. Indeed, $u^2_{500}$ shows an initial phase where the therapy is completely turned off, and the tumor can grow freely. The rationale behind this choice is to increase the size of the sensitive cells in the first part of the therapy to further promote the fight for resources between the two subpopulations. In fig. 5 we show the progression of the disease when adopting the computed controls $u^1_{500}$ and $u^2_{500}$ for a tumor with the same parameters as in fig. 1.

The graphs in fig. 5 reflect the different qualitative behavior of the computed minimizers of $\mathcal {J}^1$ and $\mathcal {J}^2$. Namely, on the one hand, we notice that the picture related to $\mathcal {J}^1$ (left) is almost indistinguishable from the progression under the MTD approach (see the picture on the left-hand side in fig. 1). On the other hand, the control obtained by minimizing $\mathcal {J}^2$ (right) succeeds in delaying the growth of the resistant subpopulation. However, in the first part of the treatment, the total tumor size exceeds the progression threshold. Hence, it turns out that for such a strategy $\tau _{TTP}^\theta < \tau _{TTP'}^\theta$ (see eq. (3.8) and (3.9) for the definitions). Indeed, when the ‘progression size-level’ is crossed for the first time, the tumor is mainly made of sensitive cells, which are killed at a proper later stage. Finally, we observe that the computed controls for the minimax formulation (i.e., related to $\mathcal {J}^1_{\max }, \mathcal {J}^2_{\max }$) show in both cases an initial phase where they are close to 1, with only minor differences between them. For this reason, these scheduling are expected to behave similarly as the MTD regime.

In Table 6 we report the TTPs resulting from the computed schedules. We insist on the fact that for having a fair evaluation of the TTP, in the rows marked as ‘Hyperbolic’ (corresponding to the cost $\ell ^2$), we used $\tau _{TTP'}^\theta$. Moreover, when testing the policy related to the linear cost $\ell ^1$, we observed $\tau _{TTP}^\theta = \tau _{TTP'}^\theta$ for every $\theta \in \Theta _N$.

**Table 6:** Strategies related to linear and hyperbolic cost.

	$n_0$	max TTP	min TTP	mean TTP
Lin. (averaged)	0.25	521 days	109 days	210 days
Hyp. (averaged)	0.25	672 days	9 days	102 days
Lin. (minimax)	0.25	506 days	109 days	209 days
Hyp. (minimax)	0.25	499 days	107 days	210 days
Lin. (averaged)	0.50	593 days	155 days	270 days
Hyp. (averaged)	0.50	850 days	15 days	336 days
Lin. (minimax)	0.50	584 days	155 days	267 days
Hyp. (minimax)	0.50	580 days	155 days	267 days
Lin. (averaged)	0.75	747 days	283 days	410 days
Hyp. (averaged)	0.75	968 days	507 days	660 days
Lin. (minimax)	0.75	752 days	282 days	402 days
Hyp. (minimax)	0.75	755 days	278 days	400 days

Comparison in terms of Time-to-Progression (TTP) between the schedules obtained by minimizing $\mathcal {J}^1, \mathcal {J}^1_{\max }$ (linear) and $\mathcal {J}^2, \mathcal {J}^2_{\max }$ (hyperbolic). The mean TTP is computed by taking the average over the elements of the set $\Theta _N$ (see eq. (3.6)). We insist on the fact that in the rows marked as ‘Hyperbolic’ we used $\tau _{TTP'}^\theta$

**Fig. 6:**
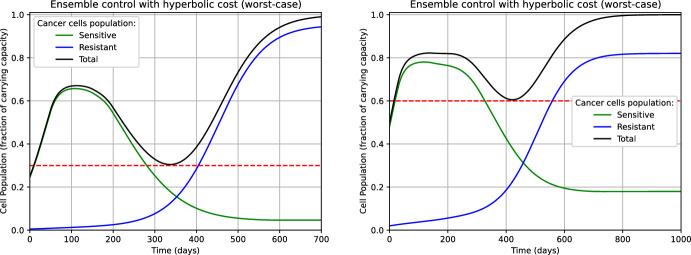
Worst-case tumors for the schedules computed by minimizing $\mathcal {J}^2$ for $n_0=0.25$ ($\tau ^\theta _{\textrm{TTP}'} = 9$ days, left) and $n_0=0.50$ ($\tau ^\theta _{\textrm{TTP}'} = 14$ days, right). The tumors corresponding to these graphs have, respectively, parameters $\theta =(1.5,0,0.72,0.02)$ (left) and $\theta =(1.5,0,0.84,0.04)$ (right). (color figure online)

The results in Table 6 confirm that the schedule obtained by minimizing $\mathcal {J}^1$ (linear cost) is substantially equivalent to MTD since the measured TTPs are almost identical. The same observation holds as well for the controls related to the minimax functionals $\mathcal {J}^1_{\max },\mathcal {J}^2_{\max }$. As for the policy related to $\mathcal {J}^2$ (hyperbolic cost), interpreting the results is more complicated. Indeed, on the one hand, for $n_0=0.50$ and $n_0=0.75$ the computed mean TTPs (336 days and 660 days, respectively) are promising and show an apparent improvement when compared to the benchmark strategy ‘On-Off’ AT (see Table 4). On the other hand, for $n_0=0.25$, the policy related to $\mathcal {J}^2$ performs poorly since the mean TTP has more than halved if compared to the other treatment approaches. In addition, for $n_0=0.25$ and $n_0=0.50$, we observe a dramatic drop in min TTP, i.e., the time-to-progression for the worst-case tumor. In fig. 6 we reported the evolution of the tumors with $n_0=0.25$ and $n_0=0.50$ for which $\tau _{\textrm{TTP}'}^\theta$ attains the minimal value (9 days and 15 days, respectively).

We notice that the dynamics of the total tumor population is qualitatively similar in the two graphs in fig. 6. Namely, the computed policy prescribes an initial phase where the tumor can freely grow. However, differently from what is observed in the right-hand side picture of fig. 5, the total population size never shrinks below the progression threshold.

**Ensemble optimal control: conclusions**

When solving ensemble optimal control problems for designing drug schedules, the choice of the cost that penalizes the tumor size plays a crucial role. On the one hand, the linear penalization led to outcomes substantially identical to MTD. On the other hand, the hyperbolic cost introduced in eq. (3.11) showed promising results. In the latter case, the strategy is to increase the number of sensitive cells in the early stage of therapy to accentuate the fight for resources in the two subpopulations. A limitation of the resulting optimal policy is that it allows the tumor to grow substantially beyond the progression threshold before initiating any reduction in tumor size (see the right-hand side of fig. 5). Moreover, in some scenarios, even during the active treatment phase, the tumor size does not decrease below the progression threshold (see fig. 6). Motivated by these observations, we propose a variant of Adaptive Therapy in the next subsection.

### ‘Off-On’ Adaptive Therapy

In this part, we suggest a variant for the ‘On-Off’ AT detailed in Subsection 3.2. The source of inspiration is the policies related to the hyperbolic cost that have been discussed in Subsection 3.3. Namely, we aim to formulate an adaptive therapy that allows tumor growth to be controlled at the early stage of the therapy, without exceeding the progression threshold. More precisely, we define the ‘Off-On’ AT through the following steps, for every $\theta \in \Theta _N$:

1. At $\tau =0$, set $u=0$ and keep it constant while $s^\theta (\tau ) + r^\theta (\tau )< 1.2\, n_0$ (vacation period).
2. After observing $s^\theta (\tau )+r^\theta (\tau )\ge 1.2\, n_0$, reset $u=1$ and keep it constant while $s^\theta (\tau ) + r^\theta (\tau )> n_0/2$ (treatment period).
3. After observing $s^\theta (\tau ) + r^\theta (\tau ) \le n_0/2$, reset $u=0$ and go to Step (1).

**Fig. 7:**
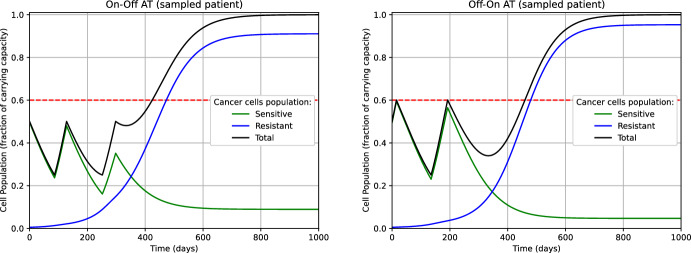
Dynamics of the tumor corresponding to $\theta = (1.5,0,0.66,0.01)$ and with initial size $n_0=0.5$ using ‘On-Off’ AT (left) and ‘Off-On’ AT (right). The dashed horizontal line represents the threshold tumor size related to the condition ‘cancer in progression’. The TTP of ‘On-Off’ AT and ‘Off-On’ AT is 424 days and 459 days, respectively, with a progression delay of 5 weeks. (color figure online)

The initial ‘vacation period’ allows the reproduction of sensitive cells to promote an increased fight for resources with the resistant clones. However, as soon as the tumor size gets close to the progression threshold, the therapy is started. Finally, the therapy is discontinued when the total population shrinks below the $50\%$ of the initial size $n_0$. We present the results in Table 7, and we show the progression of the disease for a specific parameter $\theta \in \Theta _N$ in fig. 7. We report again the information about ‘On-Off’ AT to facilitate the comparison.

**Table 7:** ‘Off-On’ Adaptive Therapy

	$n_0$	max TTP	min TTP	mean TTP
‘On-Off’ AT	0.25	575 days	109 days	213 days
‘Off-On’ AT	0.25	589 days	107 days	217 days
‘On-Off’ AT	0.50	764 days	155 days	285 days
‘Off-On’ AT	0.50	822 days	167 days	306 days
‘On-Off’ AT	0.75	1196 days	283 days	462 days
‘Off-On’ AT	0.75	1500 days	400 days	589 days

Comparison in terms of Time-to-Progression (TTP) between ‘On-Off’ AT and ‘Off-On’ AT. The mean TTP is computed by taking the average over the elements of the set $\Theta _N$ (see eq. (3.6))

The outcomes of ‘Off-On’ AT seem promising, as it shows an improvement in almost every performance indicator compared to ‘Off-On’ AT. The only exception is $\min$ TTP for $n_0=0.25$, where, in the worst-case tumor, the progression occurs 2 days earlier with ‘Off-On’ AT than when adopting ‘On-Off’ AT.

#### Remark 10

In the idealized setting considered here, waiting until the threshold $s^\theta (\tau )+r^\theta (\tau )\ge 1.2\, n_0$ is crossed increases the effect of resource competition exerted by sensitive cells on resistant ones, thereby delaying overall tumor progression, independently of baseline tumor size. This threshold should not be interpreted as a direct clinical recommendation: in patients with high baseline tumor burden, early intervention may be required. For prostate cancer, our analysis is consistent with low-burden or biochemical recurrence scenarios, where treatment initiation is guided by PSA dynamics (e.g., EMBARK trial criteria Freedland et al. 2023).

## Conclusions

From a theoretical standpoint, the main contribution of this work is the development of an ensemble optimal control framework in a general control-affine setting, which is sufficiently flexible to encompass a wide class of cancer models. In particular, it accommodates multi-species (e.g., partially resistant or tolerant subpopulations) tumor dynamics with inter-species interactions, as well as control-dependent transition mechanisms that naturally encode therapy-induced effects. Moreover, the formulation is compatible with pharmacological refinements that decouple drug dose from drug concentration through additional dynamical equations, thereby moving beyond the assumption of direct proportionality between administration and effect.

A natural limitation of the present theoretical framework lies in the restriction to a fixed finite time horizon $T>0$. In this context, it would be particularly relevant to investigate an ensemble *time-optimal* control problem, where the objective is directly related to clinically meaningful quantities such as the ‘time to progression’. This direction appears especially promising in view of applications in long-term disease management. Further extensions include the study of singular arcs (see Aronna et al. 2025a) and turnpike-type properties in the ensemble setting (see Hernández et al. 2024 for parameter-dependent problems, and Trélat and Zuazua (2025) for a survey), building on recent developments in optimal control theory.

In the computational part, we focused on a two-population model for prostate cancer. We recall that, after observing biochemical recurrence of prostate carcinoma without radiological evidence of metastasis, the choice between immediate or delayed starting of hormone therapy remains an open question to the present day. Adopting an active surveillance strategy with periodic clinical, instrumental, and laboratory reassessment of the disease remains a viable option. On the one hand, in clinical practice, this strategy is mainly applied in cases of indolent disease with long waiting time before biochemical recurrence after radical treatment ($>18$ months), long time to PSA doubling ($>12$ months), low Gleason score at diagnosis (6 or 7), and PSA levels $<1$ ng/mL post-prostatectomy or $<2$ ng/mL post radiotherapy (Karim et al. 2025; Preisser et al. 2024). On the other hand, in the setting of metastatic disease, immediate initiation of systemic treatment remains a cornerstone of cancer therapy.

The results obtained in Subsection 3.3 for the hyperbolic cost suggest that the active surveillance (already used in clinical practice) can be a nearly-optimal strategy for the long-term management of the disease within the proposed modeling framework. However, this conclusion should be interpreted in light of the simplifying assumptions that underlie the model in eq. (3.3), which do not explicitly account for more complex biological mechanisms such as resistance development, phenotypic plasticity, or spatial heterogeneity.

In this part, we have focused on a two-population model calibrated using parameter estimates reported in the literature for prostate carcinoma (non-metastatic and castration-sensitive, m0CSPC) treated with androgen deprivation therapy (ADT). While this setting provides a clinically relevant testbed for the proposed methodology, it should be understood as a simplified instance of the broader class of models covered by the theoretical framework, rather than a complete description of the underlying biological complexity.

The ‘Off-On’ Adaptive Therapy proposed in Subsection 3.4 is motivated by the structure of the optimal controls obtained in the ensemble problem with hyperbolic cost, which suggests a delayed initiation of treatment. However, in contrast to the purely optimal ensemble policy, the proposed strategy retains a clinically interpretable rule in which therapy is triggered by tumor burden, after an initial surveillance phase. Although the computational study is performed in the context of prostate cancer treated with ADT, mainly due to the availability of parameter estimates in the literature, the Off-On structure is not disease-specific and can in principle be applied to other settings where competition between sensitive and resistant populations is present. From a clinical perspective, the strategy aims at improving the ‘time to progression’ compared to standard maximum tolerated dose protocols, and in our simulations it also outperforms the previously proposed On-Off AT. A relevant limitation is that the possibility of safely delaying treatment initiation must ultimately be assessed on a case-by-case basis by clinicians, even if such a delay appears advantageous from the modeling viewpoint.

## Data Availability

Not applicable.

## Technical Proofs of Section 2

### Proof of Lemma 2.1

Let us fix $\theta \in \Theta$ and $D\in \mathcal {U}$, and, to simplify the notations, let us set $X(t):=X_D^\theta (t)$ where $X_D^\theta (t) \in \Delta ^\theta$ (see eq. (2.4)). First, arguing component by component, we notice that the system is quasi-positive: For $i\ne j$, $A_{i,j},A^I_{i,j}\ge 0$, so that, if $X_i(t)=0$, then $\dot{X}_i(t)\ge 0$. Therefore, it follows that $X_i(t)\ge 0$ for all $t\in [0,T]$.

Then, let us study $N(t):=\sum _{i=1}^n X_i(t)$. Summing the equations and using (2.3), the mutation terms cancel, yielding

$$ \dot{N}(t) = \sum _{i=1}^n \left[ r_i\Big (1-\tfrac{N(t)}{K}\Big )\Big (1-2d_i^I\tfrac{D(t)}{D_{\max }}\Big ) - d_i^T \right] X_i(t). $$

If $N(t)=K$, then $\dot{N}(t) = -\sum _{i=1}^n d_i^T X_i(t)\le 0$, so that we deduce that $N(t)\le K$ for all $t\in [0,T]$.

These two arguments show that $X_i(t)\ge 0$ for every $i=1,\ldots ,n$ and that $\sum _{i=1}^NX_i(t)\le K$, i.e., that $X(t)\in \Delta ^\theta$ for all $t\in [0,T]$. $\square$

### Proof of Lemma 2.4

In the case of $\theta \in \Theta$ fixed, the result follows directly from (Scagliotti 2023a, Proposition 2.4). However, here the thesis requires eq. (2.14) to hold uniformly as $\theta$ varies in $\Theta$. To see this, we need to investigate how the dependence on $\theta$ of the fields $F_0^\theta , F_1^\theta$ affects the estimates in the proof of (Scagliotti 2023a, Proposition 2.4). From (Scagliotti (2023a), Proposition 2.3), we first observe that, for every $\tau \in [0,T]$,

A1 $$\begin{aligned} |X_{{D+\varepsilon \delta }}^\theta (\tau ) - X_{{D}}^\theta (\tau )| \le C_1 \Vert {\delta }\Vert _{L^2} \varepsilon , \quad C_1:=3\sqrt{2} e^{\sqrt{2} L\Vert {D}\Vert _{L^2} T}, \end{aligned}$$

where $L>0$ is the Lipschitz constant of the vector fields $F_0^\theta ,F_1^\theta$. Recalling that $\Theta$ is compact and observing the smooth dependence of $\tilde{F}_0^\theta , \tilde{F}_1^\theta$ in $\theta$ (see eq. (2.1)), we conclude that $F_0^\theta ,F_1^\theta$ are uniformly Lipschitz continuous in the state variable, since they are a smooth truncation supported on $B_{3K_{\max }}(0)\subset \mathbb {R}^{n}$ of $\tilde{F}_0^\theta , \tilde{F}_1^\theta$. Hence, we conclude that eq. (A1) holds uniformly in $\theta$, which in turn implies that

$$\begin{aligned} \sup _{\theta \in \Theta } \varepsilon \left| F_i^\theta \big ( X_{{D+\varepsilon \delta }}^\theta (\tau ) \big ) - F_i^\theta \big ( X_{{D}}^\theta (\tau ) \big )\right| \le L C_1 \Vert {\delta }\Vert _{L^2} \varepsilon ^2 \qquad i=0,1. \end{aligned}$$

Finally, we need to show that there exists a modulus of continuity $\delta :\mathbb {R}_+ \rightarrow \mathbb {R}_+$ such that

$$\begin{aligned} \left| F_i^\theta (x_2) - F_i^\theta (x_1) - \frac{\partial F_i^\theta (x_1)}{\partial x}(x_1-x_2) \right| \le \delta (|x_2-x_1|) |x_2-x_1| \qquad i=0,1 \end{aligned}$$

for every $\theta \in \Theta$ and for every $x_1,x_2 \in \mathbb {R}^{n}$. However, this follows again from the smooth dependence of $\tilde{F}_0^\theta , \tilde{F}_1^\theta$ in $\theta$ and from the fact that $F_i^\theta (x) = \rho (x) \tilde{F}_i^\theta (x)$ for $i=0,1$, with $\rho :\mathbb {R}^{n}\rightarrow \mathbb {R}$ smooth and compactly supported cut-off function. Having observed that, we deduce that the estimates done in the proof of (Scagliotti 2023a, Proposition 2.4) hold uniformly in $\theta$, and we deduce the thesis. $\square$

### Proof of Lemma 2.7

Denoting for brevity with $C_{D}$ the right-hand side of eq. (2.25), we first show that $T({D},\mathcal {U})\subseteq C_{D}$. Let us consider a sequence $(\varepsilon _n)_{n\ge 1}$ such that $\varepsilon _n\rightarrow 0$ as $n\rightarrow \infty$, and let us consider $v_{\varepsilon _n} =\frac{{D}'_n- {D}}{\varepsilon _n} \in \frac{\mathcal {U}- {D}}{\varepsilon _n}$, with ${D}'_n\in \mathcal {U}$. Let us introduce

A2 $$\begin{aligned} {\begin{matrix} A_0:=\{t\in [0,T] : {D}(t)=0\}, \\ A_1:=\{t\in [0,T] : {D}(t)={D_{\max }}\}. \end{matrix}} \end{aligned}$$

Since $0\le {D}'_n\le {D_{\max }}$ a.e. and for every $n\ge 1$, it turns out that

$$\begin{aligned} {\begin{matrix} 0\le v_{\varepsilon _n}(t)\le \frac{{D_{\max }}}{\varepsilon _n} \quad \text{ a.e. } \text{ on } A_0,\\ -\frac{{D_{\max }}}{\varepsilon _n} \le v_{\varepsilon _n}(t)\le 0 \quad \text{ a.e. } \text{ on } A_1, \end{matrix}} \end{aligned}$$

which, in particular, implies that $v_{\varepsilon _n}(t)\ge 0$ and $v_{\varepsilon _n}(t)\le 0$ a.e. on $A_0,A_1$, respectively. Moreover, let $v \in L^2([0,T],\mathbb {R})$ be a $L^2$-strong cluster point of the sequence $(v_{\varepsilon _n})_{n\ge 1}$. Therefore, there exists a subsequence of $(v_{\varepsilon _n})_{n\ge 1}$ that is converging to *v* at a.e. $t \in [0,T]$, and we deduce that $v \in C_{D}$.

We now address the inclusion $C_{D} \subseteq T({D},\mathcal {U})$. Let us fix $v\in C_{D}$ and $\delta >0$. We aim at constructing $\bar{\varepsilon }>0$ and ${D}'_{\bar{\varepsilon }}\in \mathcal {U}$ such that $\Vert v- \frac{1}{\bar{\varepsilon }}({D}'_{\bar{\varepsilon }} - {D} )\Vert _{L^2}\le \delta$. To do that, we first choose $M>0$ such that $\Vert v- v_M\Vert _{L^2}\le \frac{\delta }{2}$, where $v_M\in L^2([0,T],\mathbb {R})$ is defined as the truncation of *v*, i.e., $v_M(t):=\min \left( \max \big ( v(t), -M \big ),M \right)$ for a.e. $t\in [0,T]$. Then, let us introduce $A_b:=[0,T]\setminus (A_0 \cup A_1)$, i.e., $A_b= \{ t\in [0,T]: 0<{D}(t)<{D_{\max }} \}$. Moreover, we define $A_b^k :=\left\{ t\in [0,T]: \min \big ( {D}(t),{D_{\max }}-{D}(t) \big ) \ge 1/k \right\}$ for $k\ge 2$, and we observe that

$$\begin{aligned} A_b^k\subseteq A_b^{k+1} \quad \forall k\ge 2, \quad \text{ and } \quad A_b= \bigcup _{k=2}^\infty A_b^k. \end{aligned}$$

This implies that there exists $\bar{k}\ge 2$ such that

$$\begin{aligned} \left\| v_M - v_M 1\!\!1_{A_0\cup A_1\cup A_b^{\bar{k}}} \right\| _{L^2}\le \frac{\delta }{2}, \end{aligned}$$

where $1\!\!1_B:[0,T]\rightarrow \{0,1 \}$ denotes the function that is equal to 1 on the Borel set *B*, and 0 elsewhere. Setting $A^\delta :=A_0\cup A_1\cup A_b^{\bar{k}}$ for brevity, using the triangular inequality, we notice that $\left\| v - v_M 1\!\!1_{A^\delta } \right\| _{L^2}\le \delta$. We are left to show that, setting $\bar{\varepsilon }= 1/(\bar{k} M)$, the function ${D}'_{\bar{\varepsilon }}:={D} + \bar{\varepsilon }v_M1\!\!1_{A^\delta }$ belongs to $\mathcal {U}$, i.e., $0\le {D}'_{\bar{\varepsilon }} \le {D_{\max }}$ a.e. We observe that:

- If $t \in A_0$, then by definition of $C_{D}$ we have that $v(t)\ge 0$ and $M\ge v_M(t) \ge 0$, while $1\!\!1_{A^\delta }(t)=1$. Hence, ${D}'_{\bar{\varepsilon }}(t) = \bar{\varepsilon }v_M(t) \in [0, {D_{\max }}/\bar{k}]$, and in particular $0\le {D}'_{\bar{\varepsilon }}(t) \le {D_{\max }}$.
- If $t \in A_1$, then by definition of $C_{D}$ we have that $v(t)\le 0$ and $-M \le v_M(t) \le 0$, while $1\!\!1_{A^\delta }(t)=1$. Hence, ${D}'_{\bar{\varepsilon }}(t) = {D_{\max }} + \bar{\varepsilon }v_M(t) \in [{D_{\max }}- {D_{\max }}/\bar{k}, {D_{\max }}]$, and in particular $0\le {D}'_{\bar{\varepsilon }}(t) \le {D_{\max }}$.
- If $t \in A_b^{\bar{k}}$, we have that $-M \le v_M(t) \le M$ and ${D}(t) \in [1/\bar{k}, {D_{\max }}-1/\bar{k}]$, while $1\!\!1_{A^\delta }(t)=1$. Hence, ${D}'_{\bar{\varepsilon }}(t) = {D}(t) + \bar{\varepsilon }v_M(t)\in [0,{D_{\max }}]$.
- Finally, if $t \in A_b\setminus A_b^{\bar{k}}$, we observe that $1\!\!1_{A^\delta }(t)=0$, and therefore we have ${D}'_{\bar{\varepsilon }}(t) = {D}(t)\in [0,{D_{\max }}]$ since ${D}\in \mathcal {U}$.

Since this shows the inclusion $C_{D} \subseteq T({D},\mathcal {U})$, the proof is complete. $\square$

## Footnotes

1 In hematological diseases, cancer cells typically lack strong spatial constraints and localized resource limitation, which are key drivers of ecological competition in solid tumors. However, the theoretical results in Section 2 are general and also apply to hematological malignancies, provided the underlying dynamical model can be formulated as a control-affine system of ODEs.

2 The codes used for the simulations are available at the following link: https://doi.org/10.5281/zenodo.19652678.

## Declarations

### Code availability

The codes used for the simulations are available at the following link: https://doi.org/10.5281/zenodo.19652678.
